# Development of *RS1*-specific ACMG/AMP variant classification criteria with pilot variant curation

**DOI:** 10.1007/s00439-026-02871-0

**Published:** 2026-09-04

**Authors:** Sarah Hull, M. Mero, W. Hankey, K. Lee, L. S. Sullivan, R. Ayyagari, M. D. Benson, L. Haer-Wigman, R. B. Hufnagel, H. M. J. Hussain, K. Kämpjärvi, P. A. Sieving, M. Wang, G. Wang, C. Zeitz, R. A. Lewis, R. Chen, K. C. Worley

**Affiliations:** 1 Department of Ophthalmology, Faculty of Medical and Health Sciences, University of Auckland, Auckland, New Zealand; 2 Department of Molecular & Human Genetics, Baylor College of Medicine, Houston, TX USA; 3 Department of Genetics, UNC School of Medicine, University of North Carolina, Chapel Hill, NC USA; 4 Human Genetics Center, School of Public Health, The UT Health Science Center, Houston, TX USA; 5 The Viterbi Family Department of Ophthalmology, UC San Diego Health, University of California San Diego, La Jolla, CA USA; 6 Department of Ophthalmology & Visual Science, University of Alberta, Edmonton, AB Canada; 7 Radboud University Medical Center, Nijmegen, Netherlands; 8 Center for Integrated Healthcare Research, Kaiser Permanente, Honolulu, HI USA; 9 Blueprint Genetics, Espoo, Finland; 10 Ophthalmology & Vision Science, UC Davis, Davis, CA USA; 11 Department of Ophthalmology, Gavin Herbert Eye Institute – Center for Translational Vision Research, University of California Irvine School of Medicine, Irvine, CA USA; 12 Labcorp, San Francisco, CA, USA; 13 Sorbonne Université, INSERM, CNRS, Institut de la Vision, 75012 Paris, France; 14 Department of Ophthalmology, Baylor College of Medicine, Houston, TX USA

## Abstract

Gene-based therapies are being developed for retinal diseases, including *RS1-*related X-linked retinoschisis. Therefore it is essential to determine which variants are pathogenic and which are benign when enrolling patients. The Clinical Genome Resource (ClinGen) X-Linked Inherited Retinal Diseases (XLRD) Variant Curation Expert Panel (VCEP) brings together clinician scientists, molecular biologists, and geneticists to apply their expertise and review the clinical, genetic, population, and functional evidence for variants. American College of Medical Genetics (ACMG) guidelines have been modified for *RS1* to develop a highly systematic and conservative framework for evaluating variants. The curation process involves applying 28 different codes, each with 4 strength levels (very strong, strong, moderate, supporting) across different domains of phenotype, population data, computational assessment, functional impact, and segregation. With *RS1-*specific rules, a total of 54 pilot variants were tested. These included 47 variants in ClinVar. Of these 21 variants were re-classified: 2 likely pathogenic variants and one likely benign were changed to variants of uncertain significance and 4 previously unclassified variants were changed to pathogenic, likely pathogenic and likely benign. Other changes resolved conflicts or multiple classifications.

**Supplementary Information:**

The online version contains supplementary material available at https://doi.org/10.1007/s00439-026-02871-0.

## Introduction

X-linked retinoschisis (XLRS) (MIM: #312700) due to hemizygous variants in *RS1* (MIM: *300839) is an X-linked retinal dystrophy of the inner retina (Sauer et al. 1997). Typically, it presents in the first decade of life in males with reduced vision, an electronegative electroretinogram, and a characteristic spoke-wheel appearance to the macula (George et al. 1995). This appearance arises from splitting of the inner retinal layers in the central part of the retina critical for detailed vision. Unlike other X-linked retinal dystrophies, it manifests only very rarely in females (Kirkby et al. 2023). *RS1* encodes retinoschisin, a 225 amino acid protein expressed exclusively in the retina by photoreceptors (Grayson et al. 2000). Despite being a soluble, secreted protein, its main function is cell adhesion with disulfide bonds between cysteine residues, forming a homo-octamer ring structure (Tolun et al. 2016). The majority of pathogenic variants in *RS1* are missense variants located in the discoidin domain encoded by exons 3–6 of this 6 exon gene, but truncating and splicing variants have also been reported (Molday et al. 2012). Gene-specific therapy has entered phase 1/2 trials (ClinicalTrials.gov identifiers: NCT02317887, NCT05878860, NCT02416622, NCT06066008, NCT06345898, last accessed 6th April 2026) and accurate variant classification is imperative for patient selection.

The X-linked Retinal Dystrophy Variant Curation Expert Panel (XLRD VCEP) under the governance of the Clinical Genomics Resource (ClinGen) has developed gene-specific rules for curating variants in *RS1* (Rehm et al. 2015). The framework for these rules was derived from the guidelines of the American College of Medical Genetics and Genomics and the Association for Molecular Pathology (ACMG/AMP) which are refined regularly by the ClinGen Sequence Variant Interpretation (SVI) Working Group (Richards et al. 2015; Biesecker et al. 2024). Development and refinement of these rules are described in this study. In addition, these rules were applied to a pilot curation of 54 variants.

## Methods

The curation process initially involved determining which of the 28 different codes were applicable to *RS1*. The included codes were then applied each with 4 strength levels (very strong, strong, moderate, and supporting) across different domains of computational assessment, functional impact, population data, phenotype, and segregation.

Expert biocurators adapted each code according to gene-specific rules with conflict, further refinement, and ultimate approval achieved within the XLRD VCEP bi-monthly online meetings. These online meetings included biocurators, clinician scientists, molecular biologists, and geneticists from across the world.

Rules were scored and combined to classify variants into pathogenic (P), likely pathogenic (LP), uncertain significance (VUS), likely benign (LB), or benign (B). Once rules were agreed upon, a pilot list of 54 variants were interrogated by an expert biocurator, which involved assimilating evidence from published literature, population databases and computational bioinformatic predicting tools, as well as contacting authors of published reports when further information was needed to achieve criterion threshold. The pilot variants included a list of 47 variants from ClinVar (http://www.ncbi.nlm.nih.gov/clinvar/) to determine how many could be re-classified according to these gene-specific rules (Landrum et al. 2014). Other variants were ascertained from published literature. Pilot variant curations were presented to the XLRD VCEP for classification approval, which also provided opportunity for further refinement of criteria rules. The pilot rules and variants were then submitted to ClinGen for approval.

Variants in *RS1* were referenced by their position in the transcript (GenBank: NM_000330.4); coordinates of variants were reported relative to nucleotide position 1 corresponding to the A of the ATG initiation codon. Alignment and variant calling used the GRCh38 reference genome. Variant nomenclature was assigned according to the Human Genome Variation Society (HGVS) guidelines (den Dunnen et al. 2016) (https://www.hgvs-nomenclature.org/)(http://www.varnomen.hgvs.org/). Population frequencies were derived from gnomAD (Karczewski et al. 2020) v4.1.0 accessed at https://gnomad.broadinstitute.org/.

Comparative analysis of 11 in-silico tools to predict pathogenic *RS1* variants was performed to determine which tool would perform best for *RS1*. A total of 42 pathogenic and 24 benign annotated ClinVar variants were collected from three publicly available databases: ClinVar, Leiden Open Variation Database and Human Gene Mutation Database (HGMD, v,12-20-2016). Receiver operating characteristic (ROC) curve analysis and area under the curve (AUC) scores were investigated as previously described (Brock et al. 2024). Full methods are provided in Supp Fig. 1.

Computational assessment was performed with REVEL (Ioannidis et al. 2016) at https://sites.google.com/site/revelgenomics/ for missense variants, and using SpliceAI (Jaganathan et al. 2019) at https://spliceailookup.broadinstitute.org/ for splicing variants.

When investigating novel missense variants at a codon with a previously described P or LP variant, the Grantham score was calculated to ensure the novel variant had the same score or higher. This score measures the physiochemical difference between wild-type and variant amino acids with greater differences predicted to alter the structure and therefore function of the protein (Grantham 1974).

## Results

Gene-specific codes were developed in an iterative process to gain consensus on the rules as listed in Table 1 (see Appendix 1 for a full description of *RS1* specific codes).

**Table 1:** Summary of codes

Criteria	Original ACMG description	Strength	Detail		Comment/Figure/Appendix
Pathogenic					
PVS1	Null variant in a gene where loss of function is a known mechanism of disease	Very strong	Nonsense/frameshift/splice site/deletion/start-loss variants in c.1 A-c.671 C (p.Met1-p.Cys223) Duplication in c.1 to c.671 (p.Met1-p.Cys223)		Use *RS1*-specific PVS1 decision tree (Figs. 1, 2 and 3) modified from Abou Tayoun et al. (2018) and incorporating splice site guidance from Walker et al. (2023).
		Strong	Frameshift, GT–AG splice, deletion, duplication or nonsense variant in c.672-c.677(p.Asp224 to p.*225)		
PS1	Same amino acid change as a previously established pathogenic variant regardless of nucleotide change	Strong	Same amino acid change as an established pathogenic variant classified by XLRD VCEP rules		SpliceAI scores for both variants should be within 10% of each other (Walker et al. 2023). For detail on applying PS1 code for splicing impact see Fig. 2 and Appendix 1 PS1 and PM5 should not both be used for missense variants
		Moderate	Same amino acid change as an established likely pathogenic variant classified by XLRD VCEP rules		
PS2	De novo (both maternity and paternity confirmed) in a patient with the disease and no family history	Very strong	Use SVI point scale for counting cases. Confirmed de novo with confirmed maternity worth 2.0 pts/proband, assumed maternity worth 1.0 pts per proband 4.0 pts needed for very strong		See Table 1 PS2_PM6, and phenotype as defined in PP4
		Strong	As above, 2.0 pts needed for strong		
		Moderate	As above, 1.0 pts needed for moderate		
		Supporting	As above, 0.5 pts needed for supporting		
PS3	Well-established in vitro or in vivo functional studies supportive of a damaging effect on the gene or gene product	Strong	Variants tested in high quality knock-out/knock-in variant mouse lines can meet PS3 strong		See Appendix 2, Functional Assays Table used for pilot
		Supporting	For most assays		
PS4	The prevalence of the variant in affected individuals is significantly increased compared to the prevalence in controls	Very strong	Requires 9 or more probands with XLRS		PM2_supporting must be met
		Strong	Requires 5–8 probands with XLRS		
		Moderate	Requires 3–4 probands with XLRS		
		Supporting	Requires 2 probands with XLRS		
PM1	Located in a mutational hot spot and/or critical and well-established functional domain (e.g. active site of an enzyme) without benign variation	Strong	Applies to variants in amino acids p.Cy40, p.Cys59, p.Cys63, p.Cys110, p.Cys142, p.Cys219 and p.Cys223		Form disulfide bridges required for structure of the retinoschisin monomer, dimers or octamers A maximum of 4 points should be given for PM1 and PP3 combined. PM1 can be combined with PM5.
		Moderate	Applies to amino acids p.Glu72, p.Trp122, p.Trp163, p.Arg200, p.Glu215		Form salt bridges that are required for higher order structure of octamers and paired octamers
PM2	Absent from controls (or at extremely low frequency if recessive) in ESP, 1000 Genomes or ExAC	Supporting	Low frequency in males in population databases using < 2.0 × 10^− 6^ for cut off		
PM3	For recessive disorders, detected in trans with a pathogenic variants	Not applicable			
PM4	Protein length changes due to in-frame deletions/insertions in a non-repeat region or stop-loss variants	Strong	Stop-loss variants		Variant must not be considered in any PVS1 criteria. Variant must also meet PM2.
		Moderate	In-frame deletion/insertions smaller than one whole exon, in a non-repetitive region		
PM5	Novel missense change at an amino acid residue where a different missense change determined to be pathogenic has been seen before	Moderate	Same residue as a previously established pathogenic variant classified using these specifications (and assessed independently of PM5)		Novel change must not affect splicing, must meet PP3 and have a Grantham score equal or greater than previously published variants (Grantham score does not apply for disulfide bridge variants described in PM1) PS1 and PM5 should not both be used for missense variants
		Supporting	Same residue as a previously established likely pathogenic variant classified using these specifications (and assessed independently of PM5)		
PM6	Assumed de novo, but without confirmation of paternity and maternity	Combined with PS2 for de novo data			See Table 1 PS2_PM6
PP1	Co-segregation with disease in multiple affected family members in a gene definitively known to cause the disease	Strong	Requires ≥3 meioses in 1 or 2 families		Only affected relatives with the same variant identified in the proband should be counted as segregations
		Moderate	Requires 2 meioses in a family (e.g. 2 brothers and mother genotyped; 3 brothers; uncle and nephew)		
		Supporting	Requires 1 meiosis in a family		
PP2	Missense variant in a gene that has a low rate of benign missense variation and where missense variants are a common mechanism of disease	Not applicable			
PP3	Multiple lines of computational evidence support a deleterious effect on the gene or gene product (conservation, evolutionary, splicing impact, etc.)	Strong	Applies to missense variants with a REVEL score > 0.931		As long as variants not predicted to disrupt splicing (SpliceAI score < 0.2) For awarding PP3 for splice variants use the SpliceAI flowchart (Fig. 2)
		Moderate	Applies to missense variants with a REVEL score between 0.773–0.931		
		Supporting	Applies to missense variants with a REVEL score between 0.664 and 0.772		
PP4	Patient’s phenotype or family history is highly specific for a disease with a single genetic etiology	Moderate	Highly specific: male proband diagnosed with XLRS by the age of 13 with visual acuity impairment, showing schisis, and having retinal detachment.		
		Supporting	Specific: male proband diagnosed with XLRS by the age of 13 with visual acuity impairment and showing schisis.		
PP5	Reputable source recently reports variant as pathogenic, but the evidence is not available to the laboratory to perform an independent evaluation	Not applicable			As recommended by the ClinGen SVI VCEP Review Committee (Biesecker et al. 2018).
Benign					
BA1	Allele frequency is above 5% in ESP, 1000 Genomes or ExAC	Stand alone	Allele frequency ≥ 2 × 10^− 4^ in males in population databases in the subpopulation with the highest frequency		Any variant meeting both BA1 and PP3 must be reviewed by the XLRD VCEP
BS1	Allele frequency is greater than expected for disorder	Strong	Allele frequency ≥2 × 10^− 5^ in males in population databases		Overall allele frequency in all gnomAD hemizygotes should be used.
BS2	Observed in a healthy adult individual for a recessive (homozygous), dominant (heterozygous) or X-linked (hemizygous) disorder, with full penetrance expected at an early age	Strong	Only count males over age 30 with a documented eye exam without foveo-macular schisis or atrophy Must be present in ≥3 unaffected males to meet		
BS3	Well-established in vitro or in vivo functional studies show no damaging effect on protein function or splicing	Not applicable			Insufficient benign variants tested in secretion assays to meet the threshold described in Brnich et al. (2019).
BS4	Lack of segregation in affected members of a family	Supporting	Paternal inheritance is inconsistent with this gene. For *RS1*, unaffected males over age 10 years who have been examined could be used to establish this code if they have no foveo-macular schisis and good visual acuity		
BP1	Missense variant in a gene for which primarily truncating variants are known to cause disease	Not applicable			
BP2	Observed in trans with a pathogenic variant for a fully penetrant dominant gene/disorder or observed in cis with a pathogenic variants in any inheritance pattern	Not applicable			
BP3	In frame-deletions/insertions in a repetitive regions without a known function	Not applicable			
BP4	Multiple lines of computational evidence suggest no impact on gene or gene product (conservation, evolutionary, splicing impact etc.)	Stand Alone	Missense variants: REVEL ≤ 0.003		SpliceAI ≤ 0.1
		Strong	Missense variants: REVEL 0.004–0.016		
		Moderate	Missense variants: REVEL 0.017–0.183		
		Supporting	Missense variants: REVEL 0.184–0.290 or synonymous variants or noncoding variants		
BP5	Variant found in a case with an alternate molecular basis for disease	Not applicable			
BP6	Reputable source recently reports variant as benign, but the evidence is not available to the laboratory to perform an independent evaluation	Not applicable			As recommended by the ClinGen SVI VCEP Review Committee (Biesecker et al. 2018).
BP7	A synonymous variant for which splicing prediction algorithms predict no impact to the splice consensus sequence nor the creation of a new splice site AND the nucleotide is not highly conserved	Supporting	Synonymous variants or noncoding variants with no impact on splicing (SpliceAI ≤ 0.1) AND PhyloP < 0.1 for conservation		

Key: ACMG, American College of Medical Genetics; IRD, inherited retinal disease; XLRD, X-linked Retinal Dystrophy; VCEP, Variant Curation Expert Panel; ClinGen, Clinical Genome Resource; SVI, sequence variant interpretation; ESP, exome sequencing project; ExAC, Exome Aggregation Consortium; ERG, electroretinogram

The XLRD VCEP adopted a point-based scoring system for combining the codes with values as defined as Tables 2 and 3 in Tavtigian et al.(Tavtigian et al. 2020). This system was used instead of the original ACMG rules for combining criteria that relied on a quantitative Bayesian formulation (Richards et al. 2015). Briefly, the pathogenic point values assigned for different evidence strengths were 0 (indeterminate), 1 (supporting), 2 (moderate), 4 (strong) and 8 (very strong) whereas benign point values assigned were 0 (indeterminate), -1 (supporting), -2 (moderate), -4 (strong) and − 8 (very strong). These point values were then combined to categorize a variant as P (≥10), LP (6–9), VUS (0–5), LB (-1 to -6) and B (≤-7).

Of the 28 codes, 6 were used as is, 10 were not applicable to the *RS1* gene, and 12 were adapted with *RS1-*specific modifications as described below and as shown in Table 1 and Appendix 1.

### Computational and predictive data (PVS1, PS1, PS3, PM1, PM4, PM5, PP3, BP4, BP7)

#### PVS1 decision tree

**Fig. 1:**
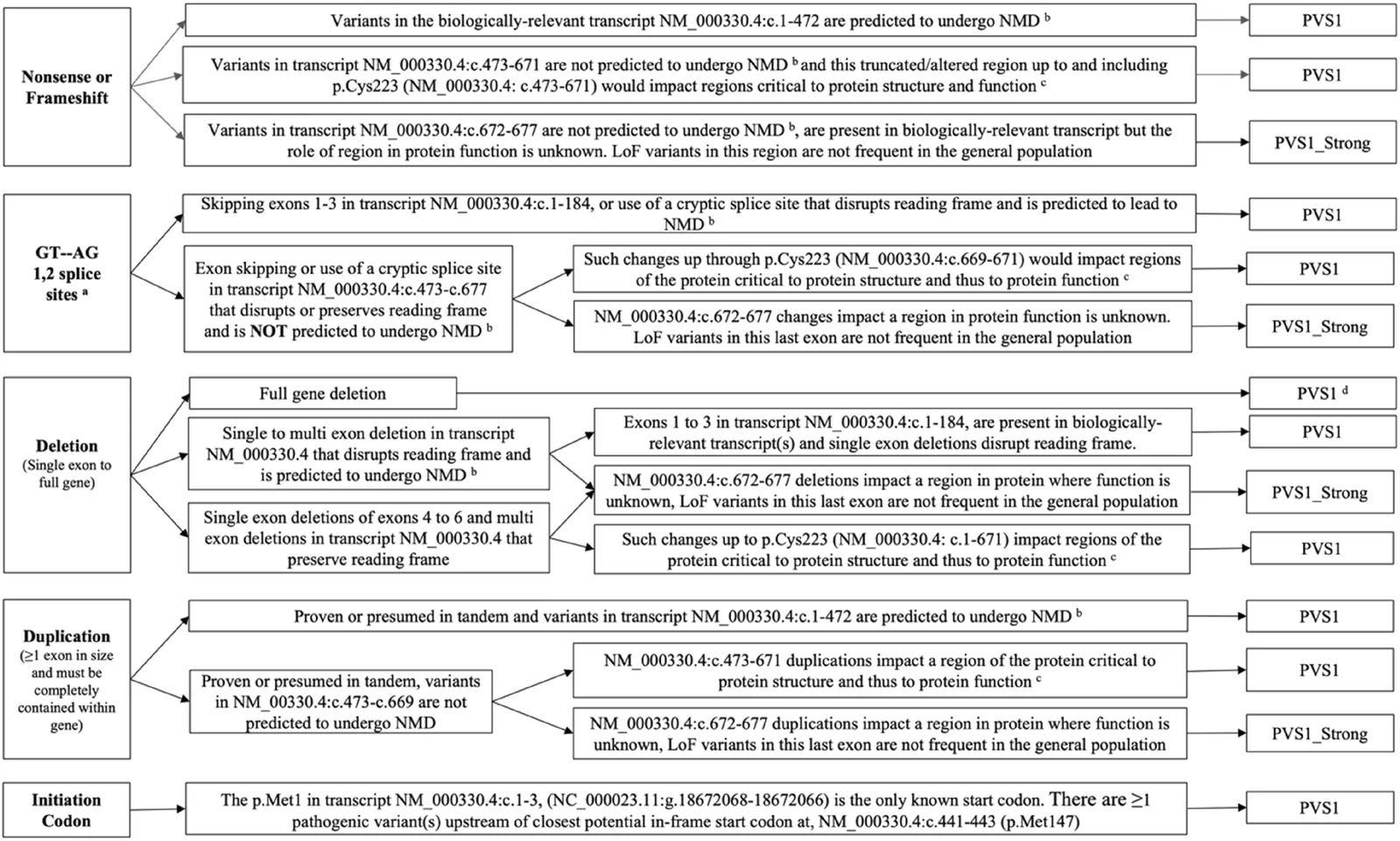
*RS1* PVS1 decision tree: adapted from Sequence Variant Interpretation recommendation for PVS1 interpretation (Abou Tayoun et al. 2018). Notes: a) This criterion should not be applied in combination with in silico splicing predictions (PP3). Additionally, splice site variants must have no detectable nearby (+/- 20nts) strong consensus splice sequence that may constitute in-frame splicing; (b) Nonsense Mediated Decay (NMD) prediction is based on the premature termination codon not occurring in the 3’most exon or in the 3’ most 50 bp of the penultimate exon (p.Ser2-p.Asp158 in transcript NM_000330.4:c.4-472, NC_000023.11:g.18,672,065 − 18,644,575); (c) Relevant domain indicated by experimental evidence proving a critical role of the domain and/or presence of non-truncating pathogenic variants in the region (NM_000330.4:c.1-671, include the discoidin domain, and specific residues involved in forming disulfide bridges and salt bridges that are important for protein structure). (d) For a full gene deletion of a known haploinsufficient gene, a pathogenic classification is warranted (in the absence of conflicting data) even though application of PVS1 alone would not reach a pathogenic classification using the combining rules in Richards et al. (2015). For a full gene deletion of *RS1* in a male, a pathogenic classification is warranted. LoF indicates loss of function

**Fig. 2:**
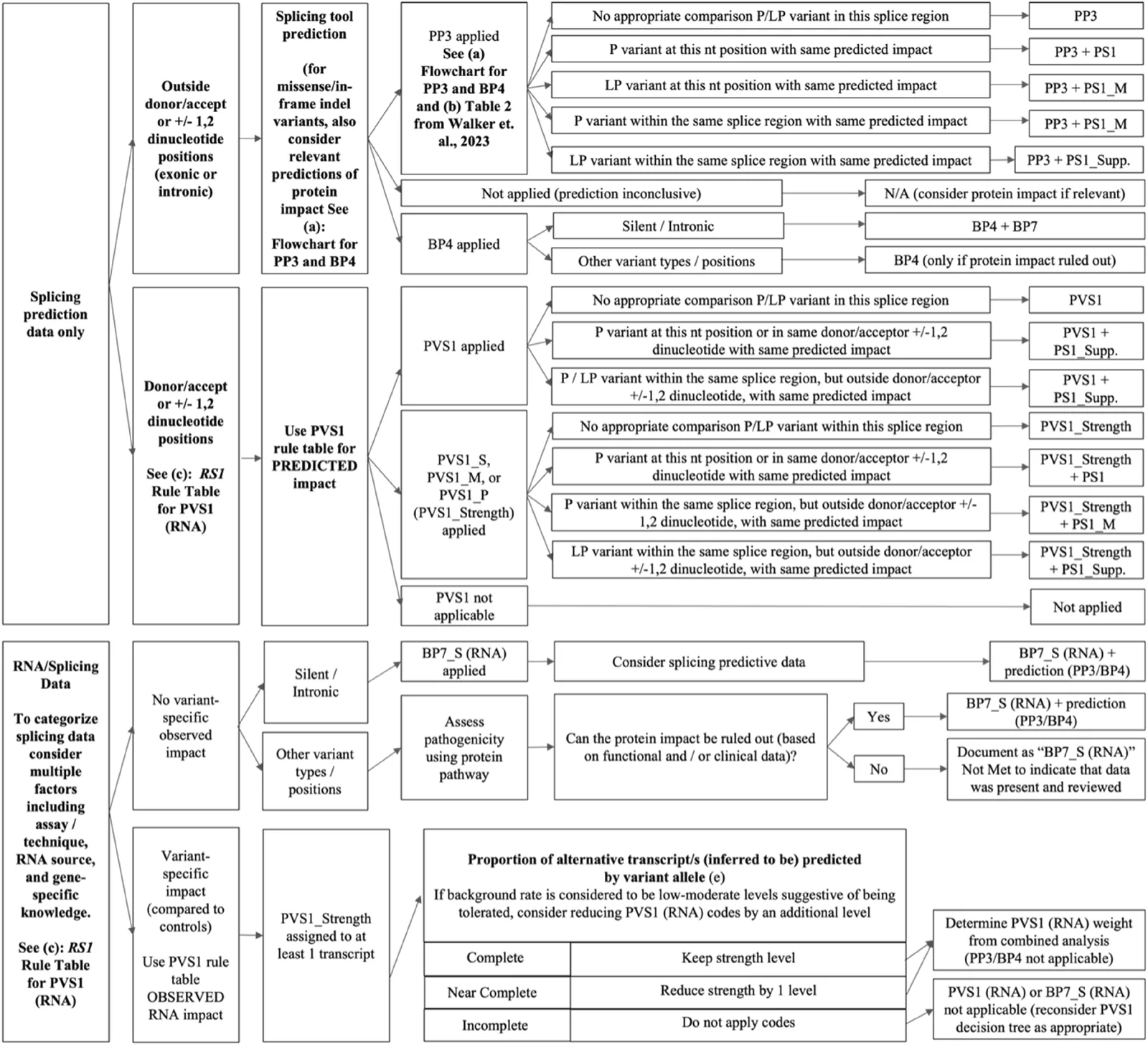
*RS1* PVS1 (RNA) decision tree for splicing: adapted from Walker et al.(Walker et al. 2023)

**Fig. 3:**
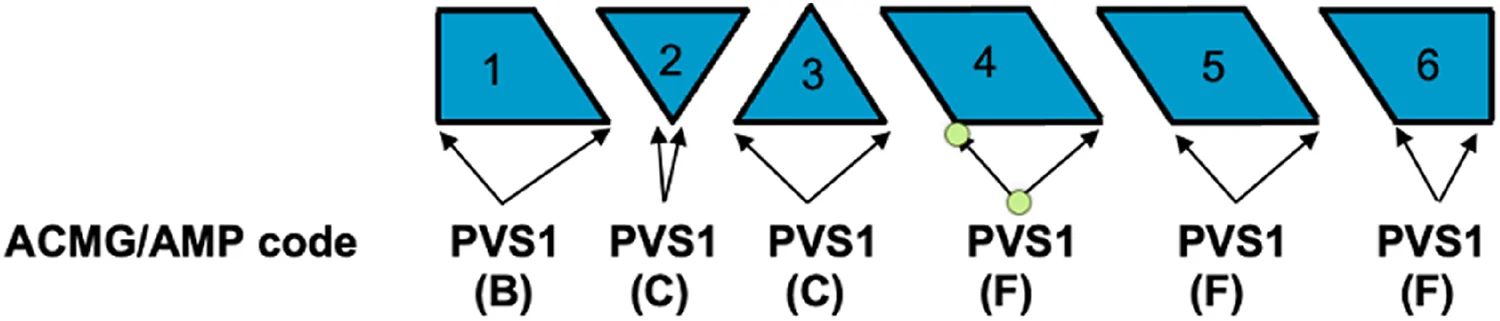
RS1 Exon Map. The retinal-specific transcript NM_000330.4 is shown with exons as blocks. Splice sites for exons 1 to 6 are indicated by arrows. The overhang shown at the top is a two-nucleotide overhang, and overhang shown on the bottom is a one-nucleotide overhang. Parallel lines represent in-frame junctions. This is adapted from Walker et al.(2023) with three modifications: Pathogenic missense variants have been identified in exons 1–6 which are all considered to be “critical to protein function”. Second, the ATG initiation site is located in exon 1 so the 5’ UTR recommendation (A) does not apply. Third, there are no known potential “rescue isoforms”. Recommendations that do apply include (B) Exon skipping or use of a cryptic splice motif eliminates the initiation codon and there are no alternative start codons, (C) Exon skipping or use of a cryptic splice motif disrupts reading frame and is predicted to undergo NMD, and (F) Exon skipping or use of a cryptic splice motif preserves reading frame and removes a region which has been established as critical to protein function.

A PVS1 decision tree was created to outline in detail when PVS1 should be applied (Figs. 1, 2 and 3; Tables 2 and 3).

**Table 2:**
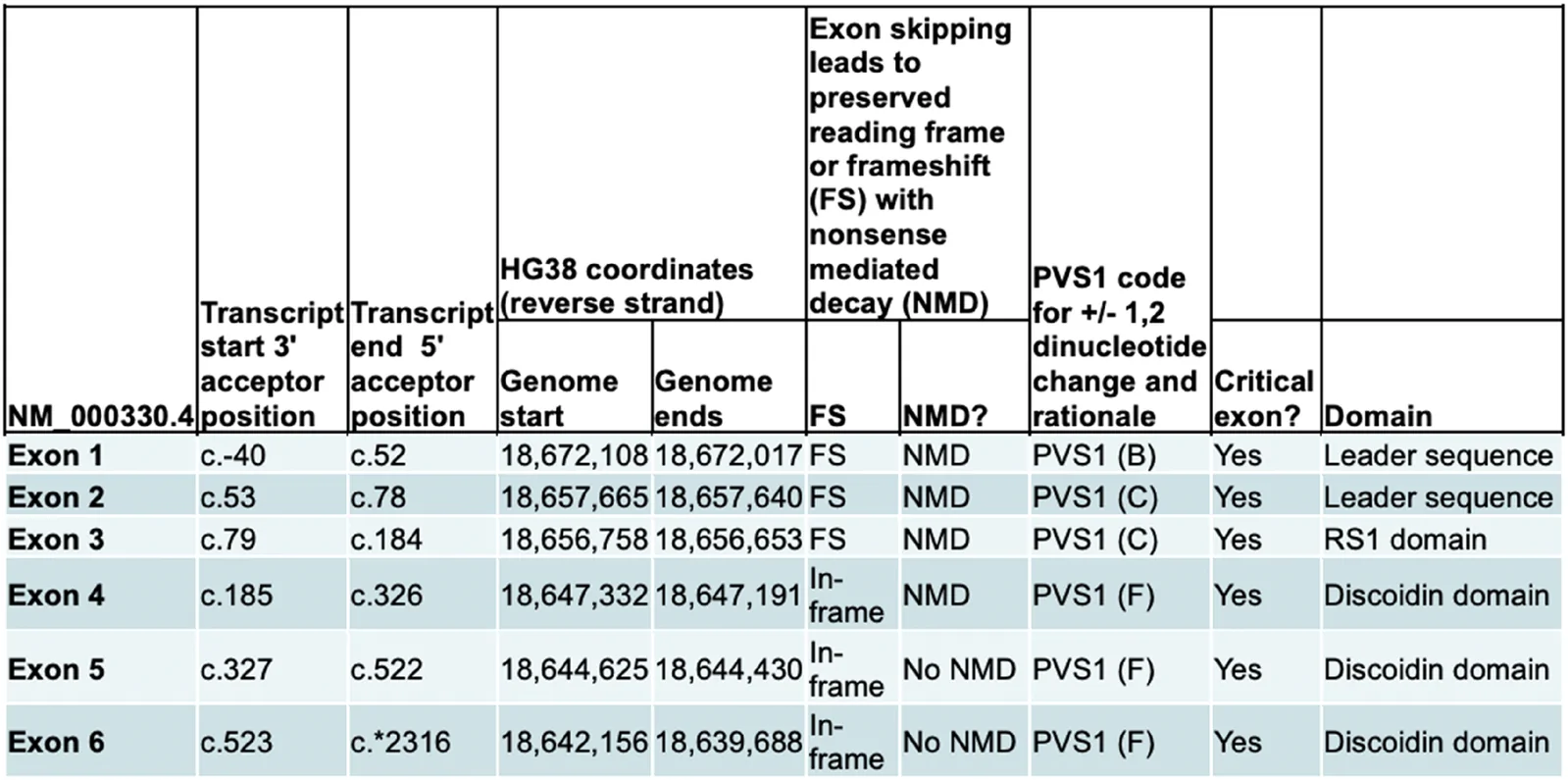
RS1 PVS1 (RNA)

Rule table for +- 1,2 changes and RNA splicing assays assessed in the transcript NM_000330.4

Based on generic gene schematic proposed by Walker et al.(2023) with the following modifications:

1. Pathogenic missense variants have been identified in exons 1–6 which are all considered to be ”critical to protein function”, thus the requirement for being more that 10% of total protein length does not apply.
2. ATG initiation site is located in exon 1 so 5’ UTR recommendation (A) does not apply.
3. No potential ”rescue isoforms” are known.
4. Use this table to assign appropriate PVS1 code and rationale.

Recommendations that do apply include (B) Exon skipping or use of a cryptic splice motif eliminates the initiation codon and there are no alternative start codons, (C) Exon skipping or use of a cryptic splice motif disrupts reading frame and is predicted to undergo NMD, and (F) Exon skipping or use of a cryptic splice motif preserves reading frame and removes a region which has been established as critical to protein function.

**Table 3:**
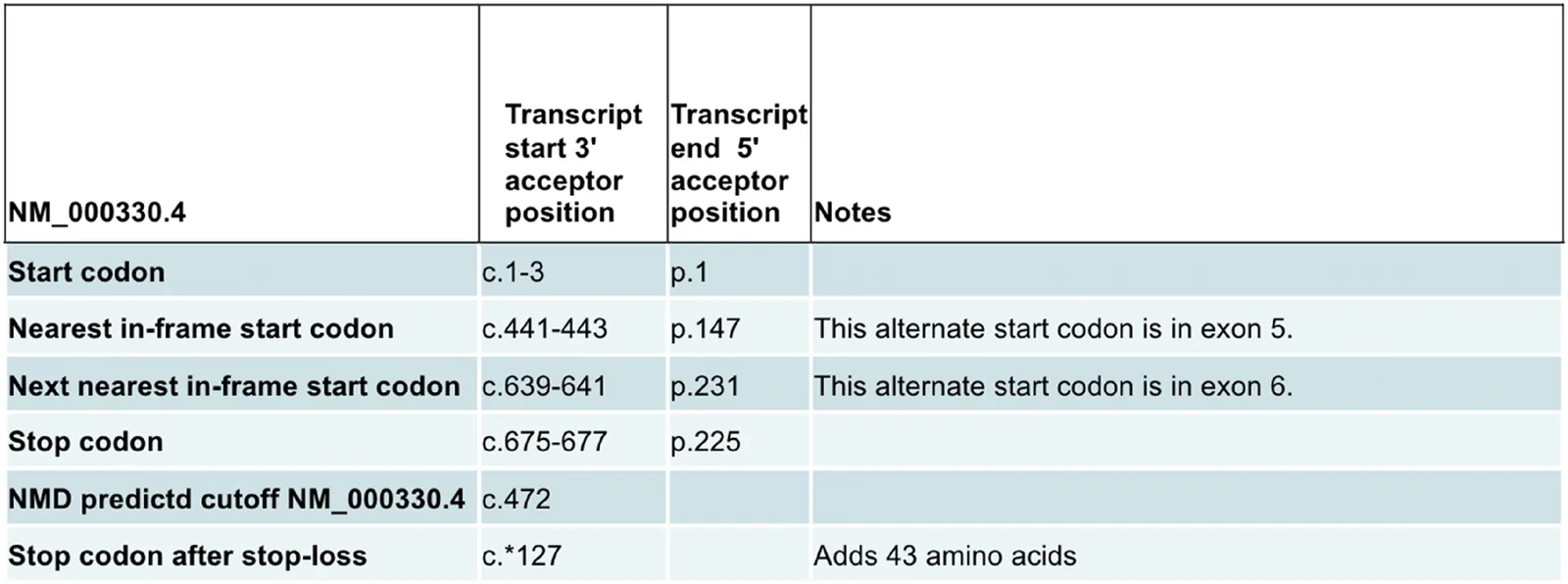
RS1 PVS1 (RNA) Feature Locations

This table defines the locations of RS1 features used to assess RNA splicing impact in the transcript NM_000330.4. Based on generic gene schematic proposed by Walker et al. (2023)

The PVS1 code for start-loss variants (i.e., missense variants in the initiating codon) was reviewed and re-classified. There was no known alternative start codon in other transcripts and multiple reports of single nucleotide variants in the first 3 nucleotides. The next in-frame methionine was found in exon 5; therefore, the panel agreed to raise the PVS1 code from moderate to very strong.

The exon schematic in Fig. 3, demonstrates the location of the splice site in each codon and the functional impact of critical regions where splicing variants are predicted to lead to exon skipping and nonsense mediated decay or loss of a critical protein functional element.

### Computational assessment

Recommended REVEL thresholds for PP3 and BP4 were derived from the ClinGen SVI working group recommendations (Pejaver et al. 2022). Comparative analysis of 11 in-silico tools to predict pathogenic *RS1* variants supported REVEL as one of the best-performing tools (Fig. 1). SpliceAI thresholds and methodology on applying codes related to splicing data in relation to PVS1, PS3, PP3, BS3, BP4, and BP7 were derived from the ClinGen SVI Splicing Subgroup recommendations (Walker et al. 2023).

### Functional impact

A PM1 code specification for *RS1* was initially not applicable. However, functional data established that 7 specific cysteine residues (p.Cys40, p.Cys59, p.Cys63, p.Cys110, p.Cys142, p.Cys219 and p.Cys223) are essential for folding, multimerization and secretion (Wu et al. 2005; Bush et al. 2016). They form disulfide bridges essential for the assembly of the retinoschisin monomer, dimer, or octamer. Interrogation of gnomAD established a lack of known benign variation with single alleles reported for each of p.Cys63Tyr and p.Cys219Gly in XX individuals (no further detail known) and the rest absent. These critical substitutions were therefore eligible for PM1_strong.

In addition, there are five residues (p.Glu72, p.Trp122, p.Trp163, p.Arg200, and p.Glu215) critical for forming a salt bridge in the interface between the two adjacent RS1 subunits in the octamer driving multimerization (Tolun et al. 2016). Interrogation of gnomAD found a lack of benign variation at these 3 residues with only one variant found in one XY individual (p.Glu72Lys, 1 in 363,121 XY individuals). Therefore these were determined to be eligible for PM1.

Variants in *RS1* could be eligible to meet both PM1 and PM5 which for some other genes would be considered mutually exclusive. This was permitted as PM1 was defined by identifying functionally critical residues distinct from PM5 rather than being defined as variants in a mutational hot spot. This allowed some variants to be awarded both codes.

Application of the PS3 code for missense variants was used for functional data either with knock-in/knock-out mouse models giving PS3_strong or cell assay studies giving PS3_supp. These data and the type of studies that represent an approved assay for the purposes of the pilot are included as Appendix 2. (Published Functional Evidence Assays for PS3/BS3). Studies frequently demonstrated secretion deficits (Wu and Molday 2003; Wang et al. 2002; Dyka and Molday 2007; Gleghorn et al. 2009).

The upgrade of the PM4 code to PM4_strong for stop-loss variants was approved based on the substantial impact these variants have on the RS1 protein structure. Specifically, a stop-loss variant results in the incorporation of 42 additional amino acids, increasing the protein length by 19%. This alteration would be expected to introduce 4 additional cysteines, likely disrupting the tight conformation of the RS1 protein, preventing the proper connection of RS1 units. Structural studies have demonstrated that other substitutions that add or remove cysteines in RS1 are deleterious, thereby justifying the application of the PM4_strong code for this variant classification (Wu and Molday 2003; Dyka and Molday 2007; Wang et al. 2006; Liu et al. 2019; Plössl et al. 2018).

### Population data (PM2, BA1, BS1)

Allele frequency of a variant was derived from gnomAD v4 1.0 with application of codes PM2/BA1/BS1 dependent on specific thresholds. These were calculated with the Whiffin/Ware calculator with thresholds of < 1.0 × 10^− 6^, ≥ 1 × 10^− 4^, and ≥1 × 10^− 5^, respectively (Whiffin et al. 2017). However, these were adapted during XLRD VCEP variant review due to the observation that some P or LP *RS1* variants (according to ClinVar) were found in multiple hemizygous individuals in gnomAD v4.1.0. This meant that some variants would not meet the previously selected threshold of < 1.0 × 10^− 6^ for PM2_Supporting. Thresholds were therefore adapted as follows:

- If absent from gnomAD, or absent from hemizygous individuals in gnomAD, or allele frequency of < 2.0 × 10^-6^ in hemizygous individuals, code PM2_Supporting was applied.
- If present with a frequency ≥2 × 10^-4^, BA1 (met) was applied.
- If present with a frequency ≥2 × 10^-5^, BS1 (met) was applied.

### Clinical data (PS2, PS4, PP1, PP4)

#### Phenotype

For any probands being considered for any pathogenic phenotype codes (PS2, PS4, PP1, PP4) at any strength, certain phenotypic characteristics were required: for a highly specific phenotype, affected males should have presented by 13 years of age with some functional vision impairment (as measured by best corrected visual acuity) observed foveo-macular schisis and retinal detachment (Hahn et al. 2022). A specific phenotype did not have retinal detachment included. Electroretinogram measurement of a subnormal B wave was considered supportive evidence. It was recognised that the age of presentation was older than the age of onset and therefore 13 years was chosen to avoid excluding reported patients incorrectly (Hahn et al. 2022). In addition, probands could be considered only if the variant met PM2_supporting.

Considering PS4 (the prevalence of the variant in affected individuals is increased substantively compared to the prevalence in controls), the strength of the code was determined by the number of probands affected:

- Very strong required ≥9 probands.
- Strong required 5–8 probands.
- Moderate required 3–4 probands.
- Supporting required 2 probands diagnosed with XLRS.

#### Segregation

For code PP1, co-segregation with disease in multiple affected family members in a gene definitively known to cause the disease; a pedigree should be included if there was no male-to-male transmission, and affected males met the specific phenotype as described above.

- PP1_Strong required ≥3 meioses in 1 or 2 families.
- PP1_Moderate required 2 meioses in a family (e.g., 2 brothers and mother genotyped; 3 brothers without mother’s genotype; nephew and maternal uncle).
- PP1_Supporting required 1 meiosis in a family (e.g., 2 brothers).

The number of meioses used was based on Table 3 in ClinGen published guidance on awarding points for co-segregations in X-linked disease and further refined recognising that XLRS is only expressed in males (Biesecker et al. 2024).

#### De novo

For PS2 (*de novo* in a patient with the disease and no family history): the code strength depended on the number of probands reported with the variant and whether maternity was confirmed or assumed (given that egg donation or adoption could be confounders). An SVI point scale for counting cases was used, and the PS2 and PM6 codes were combined (Table 1 PS2_PM6, use option 1 ‘Phenotype highly specific for gene’).

- Very strong required 4 points, 2 points awarded per proband if confirmed *de novo* with confirmed maternity, and 1 point if assumed maternity.
- Strong required 2 points.
- Moderate required 1 point.
- Supporting required 0.5 points.

#### Pilot variants

**Fig. 4:**
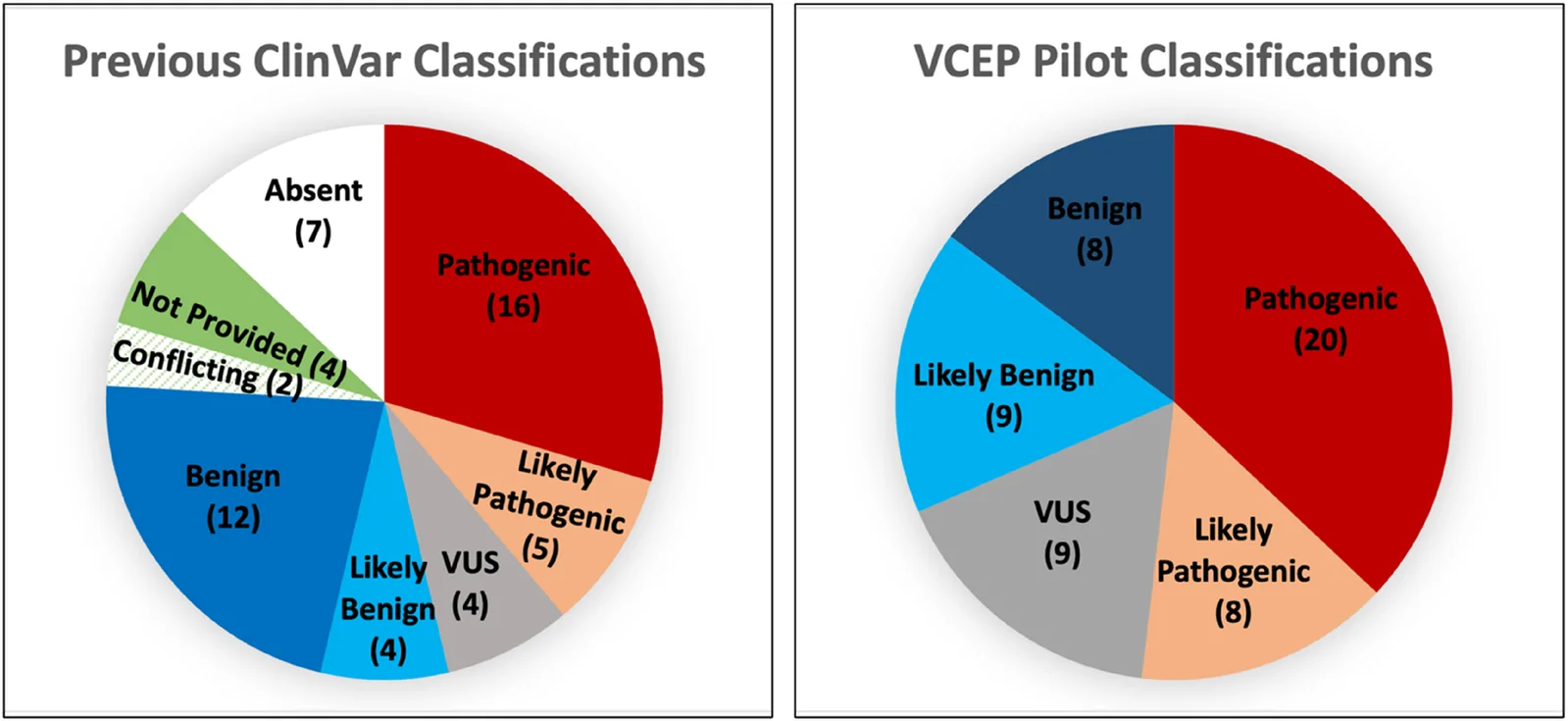
*RS1* Variant Classification Pilot Result. The 54 variants evaluated include variants found in each exon and with each classification. Pilot variants listed in ClinVar comparing expert assertion before and status after the X-linked Retinal Dystrophy Variant Curation Expert Panel (VCEP) pilot curation

A pilot list of 54 variants were curated including 47 variants already in ClinVar (Fig. 4, Table 2). Applying the *RS1* specifications altered the ClinVar Classification for 21 variants including changing 2 variants from LP to VUS, 1 variant from LB to VUS and 4 with no classification to P (2), LP (1) and LB (1) (Table 3). Two variants with conflicting data were reclassified: one with ClinVar LP/VUS to VUS and one ClinVar VUS/LB to LB. Five mixed classifications such as P/LP or B/LB were resolved into single categories. Seven reclassifications moved a variant from P to LP, LP to P or B to LB. The remaining 26 variants retained their original ClinVar classification. Of 7 variants not already in ClinVar, 4 were classified as P, 2 were classified as LP and 1 was classified as a VUS.

#### Example pilot variant curation

Variant NM_000330.4(*RS1*):c.78 + 5 G > A is an intronic variant without ClinVar submission. XLRD VCEP specific scoring rules led to a total of 6 points and a classification of LP:

- Criterion PM2: Allele frequency < 2.0 × 10-6 in hemizygous individuals in population databases. The variant is absent from hemizygous individuals in gnomAD v4 1.0. PM2_Supporting. **1 point**.
- Criterion PP3: Multiple lines of computational evidence support a deleterious effect on the gene/gene product. The splicing impact predictor SpliceAI gives a score of 0.64, which is above the ClinGen XLRD VCEP recommended threshold of ≥ 0.2 and predicts a damaging impact on splicing. PP3_met **1 point**.
- Criterion PS4: Prevalence in affected patients statistically increased over controls. This variant has been reported in at least 3 apparently unrelated probands meeting the PS4 requirements of a male diagnosed with XLRS (Xiao et al. 2023; Bender et al. 2022; Kim et al. 2009). PS4_mod **2 points**.
- Criterion PS1: Same amino acid change as a previously established P variant. This variant is in the same donor motif as NM_000330.4 (*RS1*):c.78 + 1G > A. PS1_mod **2 points**.

Variant NM_000330.4(*RS1*):c.35T > A (p.Leu12His) is a missense variant with two submissions to ClinVar (Variation ID 98946), one LP and one not provided. XLRD VCEP specific scoring rules led to a total of 3 points and classification as a VUS:

- Criterion PM2: Allele frequency < 2.0 × 10-6 in hemizygous individuals in population databases. The variant is absent from hemizygous individuals in gnomAD v4 1.0. PM2_Supporting. **1 point**.
- Criterion PS3: Well-established functional studies show a deleterious effect. COS cells exogenously expressing the variant exhibit loss of RS1 secretion into the medium relative to the wild-type control(Wang et al. 2002). PS3_Supporting **1 point.**
- Criterion PS4: Prevalence in affected patients statistically increased over controls. This variant has been reported in at least 2 apparently unrelated probands meeting the PS4 requirements of a male diagnosed with XLRS (The Retinoschisis Consortium 1998; Georgiou et al. 2022; Lenassi et al. 2020; De Silva et al. 2020; Fahim et al. 2017; Bender et al. 2022). PS4_Supporting **1 point**.
- Criteria PP3 and BP4: PP3, multiple lines of computational evidence support a deleterious effect on the gene/gene product and criteria BP4, multiple lines of computational evidence suggest no impact on the gene/gene product. The computational predictor REVEL gives a score of 0.633, which is between the ClinGen XLRD VCEP threshold of 0.664 to 0.290 and does not predict a damaging effect on RS1 function. Additionally, the splicing impact predictor SpliceAI gives a score of 0.00, which is below the ClinGen XLRD VCEP recommended threshold of ≥ 0.2 and does not strongly predict an impact on splicing. Collectively, both BP4 and PP3 codes do not apply. **0 points**.

Further example variant curations are provided as Appendix 3.

## Discussion

Gene-specific guidelines for variant curation permit highly specific and accurate rules to apply when considering pathogenicity of a variant. Development of these guidelines for *RS1* required a collaborative approach within the XLRD VCEP. Application of these guidelines to pilot variants permitted reclassification for some variants already in ClinVar. This process of variant curation is approved by the US Food and Drug Administration providing validity to these findings.

Through detailed expert curator investigation and subsequent XLRD VCEP review, the 28 different codes were modified and adapted based on all available data. These resulted in 6 being used without modification, 10 being classed as not applicable, and 12 codes adapted to ensure the rules for these codes were robust and specific.

With X-linked genes less commonly curated by ClinGen, this study provides a useful perspective on the approach to curation when variants are present on one allele only. X-linked retinoschisis is unusual compared to other X-linked retinal dystrophies, as a female phenotype has only been very rarely reported (Kirkby et al. 2023). When considering population data, hemizygous allele frequencies were specifically used. However, some variants already classified in ClinVar as P or LP were found in multiple hemizygous individuals thus exceeding the initial set threshold to be counted for PM2. These thresholds were adapted to reflect the gene specific frequencies.

Of the 47 variants already in ClinVar, 21 were reclassified. These included changing 2 variants from LP to VUS and 3 with no classification changed to P or LP. Considering eligibility for trial entry for patients with XLRS, this variant classification is particularly impactful. Two variants with conflicting data were reclassified: one with ClinVar LP/VUS to VUS and one ClinVar VUS/LB to LB.

There were initial challenges in the variant curation for missense variants not reaching sufficient points for LP or P but experimental data from electron microscopy and protein structure modelling analysis, recognized specific amino acid residues to be integral to structure and meeting criteria for PM1.

Other challenges arose from a lack of information in publications regarding clinical data, pedigrees, and age of clinical discovery for probands which reduced the number of probands that could be counted for certain codes such as PS4 or PP1. We encourage all our colleagues in the scientific community to publish detailed clinical phenotypic and segregation data and for peer-reviewers to request such data.

The use of gnomAD allele frequencies as ‘healthy’ controls has become more challenging. Version 4.0 includes 416,555 samples from the UK Biobank which did not exclude any specific phenotypes. Therefore, with affected individuals with rare diseases included, P and LP variants are likely to be increasingly identified. Awareness of this limitation when setting thresholds of frequency from population datasets is important to avoid erroneously misclassifying a variant.

In summary, *RS1-*specific guidelines for variant curation have been developed based on detailed and exhaustive evidence permitting accurate and improved codes. These have been shown in the pilot curation to be readily applicable and give a strong degree of certainty in pathogenicity prediction.

## Web resources

Clinical Trials: https://clinicaltrials.gov.

OMIM: https://omim.org.

ClinVar: http://www.ncbi.nlm.nih.gov/clinvar/.

ClinGen: https://clinicalgenome.org/.

ClinGen CSpec Registry: https://cspec.genome.network/cspec/ui/svi/.

ClinGen Sequence Variant Interface: https://clinicalgenome.org/working-groups/sequence-variant-interpretation/.

Human Genome Variation Society (HGVS): https://hgvs-nomenclature.org/http://www.varnomen.hgvs.orghttps://hgvs-nomenclature.org/.

gnomAD: https://gnomad.broadinstitute.org/.

REVEL: https://sites.google.com/site/revelgenomics/.

SpliceAI: https://spliceailookup.broadinstitute.org/.

## Supplementary Information

Below is the link to the electronic supplementary material.

Supplementary Figure 1

Appendix 1

Appendix 2

Appendix 3

Supplementary Tables 1, 2, 3

## Data Availability

Datasets generated during the current study are provided as Appendices 1-3 and Supplementary Table 2.

## Appendix

Appendix 1: Full description of *RS1* specific codes from ClinGen CSpec Registry (pdf file).

Appendix 2: Published Functional Evidence Assays for PS3/BS3 for pilot (excel file).

Appendix 3: Example variant curations (pdf file).
