# Supplementary material for: Development of RS1-specific ACMG/AMP variant classification criteria with pilot variant curation
Source: Hum Genet. 2026 Sep 4;145(1):75. doi: 10.1007/s00439-026-02871-0 (PMC13545035; doi:10.1007/s00439-026-02871-0)
Supplement: Supplementary file 1 [file 439_2026_2871_MOESM2_ESM.pdf]

Criteria Specification

ClinGen X-linked Inherited Retinal Disease Expert Panel Specifications to the ACMG/AMP Variant Interpretation Guidelines for RS1 Version 1.0.0

Affiliation: X-linked Inherited Retinal Disease VCEP
Type:
Description : X-linked IRD Expert Panel Specifications to the ACMG/AMP Variant Interpretation Guidelines for RS1.
Version : 1.0.0
Released : 5/16/2025

Rules for RS1

General Comments: In addition to the specific criteria for codes listed below, the VCEP has adopted the point-based combining scoring in Table 2 and Table 3 of PMID: 32720330. Table 2 assigns points for pathogenic (0 for indeterminate, 1 for supporting, 2 for moderate, 4 for strong, and 8 for very strong), and benign (0 for indeterminate, -1 for supporting, -2 for moderate, -4 for strong, and -8 for very strong) codes. Table 3 provides final categories of Pathogenic (>= 10), Likely Pathogenic (6-9), Uncertain (0-5), Likely Benign (-1 to -6), Benign (<=-7). This replaces the combining rules listed below.

Gene: RS1 (HGNC:10457)
Transcripts:
NM_000330.4

HGNC Name: retinoschisin 1
Disease:
X-linked retinoschisis (MONDO:0010725) **Mode of Inheritance:** X-linked inheritance

Criteria & Strength Specifications

PVS1

Original ACMG Summary

Null variant (nonsense, frameshift, canonical +/−1 or 2 splice sites, initiation codon, single or multi-exon deletion) in a gene where loss of function (LOF) is a known mechanism of disease.
Caveats:
- Beware of genes where LOF is not a known disease mechanism (e.g. GFAP, MYH7).
- Use caution interpreting LOF variants at the extreme 3’ end of a gene.
- Use caution with splice variants that are predicted to lead to exon skipping but leave the remainder of the protein intact.
- Use caution in the presence of multiple transcripts.

Very Strong

Use attached RS1-specific PVS1 Decision Tree file, which has been modified from Abou Tayoun, et al., 2018 (PMID 30192042) and incorporates splice site guidance from Walker et al, 2023 (PMID 37352859). This strength applies to nonsense, frameshift, splice site, and deletion variants in NM_000330.4 c.1A to c.671C (p.Met1 to p.Cys223) and to duplications in NM_000330.4 c.1 to c. 472 (p.Met1 to p.Asp158).

The structure of RS1 is important for function. The RS1 monomers have cystine disulfide bonds within a RS1 monomer and bonds with the two neighboring monomers to form an octomer substructure and bonds to the second RS1 octomer that completes the paired octomer structure. Within this structure there are a number of sites that have been shown to function in other types of protein structure stabilization.

Modification Type: Gene-specific

Strong

Use attached RS1-specific PVS1 Decision Tree file, which has been modified from Abou Tayoun, et al., 2018 (PMID 30192042) and incorporates splice site guidance from Walker et al, 2023 (PMID 37352859).

This strength applies to frameshift variants, GT--AG splice variants, deletions, duplications or nonsense variants in NM_000330.4 c.672 to c.677 (p.Asp224 to p.*225).

Modification Type: Gene-specific

Moderate

Use attached RS1-specific PVS1 Decision Tree file, which has been modified from Abou Tayoun, et al., 2018 (PMID 30192042) and incorporates splice site guidance from Walker et al, 2023 (PMID 37352859).

Modification Type: Gene-specific

PS1

Original ACMG Summary

Same amino acid change as a previously established pathogenic variant regardless of nucleotide change.
Example: Val->Leu caused by either G>C or G>T in the same codon.
Caveat: Beware of changes that impact splicing rather than at the amino acid/protein level.

VCEP Specifications: Refer to the RS1_-specific PVS1 Decision Tree document for more complete information.

Strong

Same amino acid change as a previously established **Pathogenic** variant. Comparison variant must have been evaluated by the X-linked IRD VCEP using these rules and established as a Pathogenic variant and classified as such. For assessing same amino acid changes, SpliceAI scores for both variants should be within 10% of each other.

When evaluating splicing impact, as a previously classified Pathogenic variant,

- Use in conjunction with PP3 for variants located outside the splice donor/acceptor +/-1,2 dinucleotide positions that have a SpliceAI score ≥0.2 and have a comparable nucleotide variant at the same position that has been designated **Pathogenic**.
- Used in conjunction with PVS1 for variants located at the splice donor/acceptor +/-1,2 dinucleotide positions and that have a comparable variant within the same splice donor/acceptor +/-1,2 dinucleotide that has been designated **Pathogenic**.

Specific combinations are found in RS1-specific PVS1 Decision Tree part (b) (Table 2 from Walker 2023).

Modification Type: Clarification,General recommendation

Moderate

Same amino acid change as a previously established **(Likely) Pathogenic** variant. Comparison variant must have been evaluated by the X-linked IRD VCEP using these rules and established as a Likely Pathogenic variant and classified as such. For assessing same amino acid changes, SpliceAI scores for both variants should be within 10% of each other.

When evaluating splicing impact, with a previously classified Likely Pathogenic or Pathogenic variant,

- Use in conjunction with PP3 for variants located outside the splice donor/acceptor +/-1,2 dinucleotide positions that have a SpliceAI score ≥0.2 and have a comparable nucleotide variant at the same position that has been designated **Likely Pathogenic**.
- Used in conjunction with PP3 for variants located outside the splice donor/acceptor +/-1,2 dinucleotide positions that have a SpliceAI score ≥0.2 and have a comparable nucleotide variant within the same splice region with the same predicted impact that has been designated **Pathogenic**.
- Use in conjunction with PVS1 at any strength for variants located within splice donor/acceptor +/-1,2 dinucleotide positions that have a SpliceAI score ≥0.2 and a comparable nucleotide variant within the same splice motif region but outside the donor/acceptor +/-1,2 dinucleotide positions with the same predicted impact that has been designated **Pathogenic**.

Specific combinations are found in RS1-specific PVS1 Decision Tree part (b) (Table 2 from Walker 2023).

Modification Type: Clarification,General recommendation

Supporting

Same amino acid change as a previously established **(Likely) Pathogenic** variant. Comparison variant must have been evaluated by the X-linked IRD VCEP using these rules and established as a Likely Pathogenic variant and classified as such. For assessing same amino acid changes, SpliceAI scores for both variants should be within 10% of each other.

When evaluating splicing impact, with a previously classified Likely Pathogenic variant,

- Use in conjunction with PP3 for variants located outside the splice donor/acceptor +/-1,2 dinucleotide positions that have a SpliceAI score ≥0.2 and have a comparable nucleotide variant variant within the same splice motif region but outside the donor/acceptor +/-1,2 dinucleotide positions with the same predicted impact that has been designated **Likely Pathogenic**.
- Used in conjunction with PVS1 for variants located within the splice donor/acceptor +/-1,2 dinucleotide positions that have a SpliceAI score ≥0.2 and have a comparable nucleotide variant at the same splice donor/acceptor +/-1,2 dinucleotide position with the same predicted impact that has been designated **Pathogenic**.
- Use in conjunction with PVS1 for variants located within splice donor/acceptor +/-1,2 dinucleotide positions that have a SpliceAI score ≥0.2 and a comparable nucleotide variant within the same splice motif region but outside the donor/acceptor +/-1,2 dinucleotide positions with the same predicted impact that has been designated either **Likely Pathogenic** or **Pathogenic**.
- Use in conjunction with PVS1 at any strength for variants located within splice donor/acceptor +/-1,2 dinucleotide positions that have a SpliceAI score ≥0.2 and a comparable nucleotide variant within the same splice motif region but outside the donor/acceptor +/-1,2 dinucleotide positions with the same predicted impact that has been designated **Likely Pathogenic**.

Specific combinations are found in RS1-specific PVS1 Decision Tree part (b) (Table 2 from Walker 2023).

Modification Type: Clarification,General recommendation

PS2

Original ACMG Summary

De novo (both maternity and paternity confirmed) in a patient with the disease and no family history.
Note: Confirmation of paternity only is insufficient. Egg donation, surrogate motherhood, errors in embryo transfer, etc. can contribute to non-maternity.

VCEP Specifications **Note that probands being considered for any pathogenic phenotype codes (e.g. PP1, PP4, PM6, PS2, PS4) at any strength must have the following phenotype characteristics: affected males should have some functional vision impairment by age 13,**

with either observed foveo-macular changes or ERG measurement of a subnormal B wave.

Very Strong

Use SVI point scale for counting cases (see Table 1 on PS2_PM6 table), use option 1 “Phenotype highly specific for gene.” Confirmed *de novo* with confirmed maternity worth 2.0 points per proband. Assumed *de novo* with assumed maternity worth 1.0 points per proband. Total 4.0 points to use at the very strong strength.

Modification Type: Gene-specific

Strong

Use SVI point scale for counting cases (see Table 1 on PS2_PM6 table), use option 1 “Phenotype highly specific for gene.” Confirmed *de novo* with confirmed maternity worth 2.0 points per proband. Assumed *de novo* with assumed maternity worth 1.0 points per proband. Total 2.0 points to use at the strong strength.

Modification Type: Gene-specific

Moderate

Use SVI point scale for counting cases (see Table 1 on PS2_PM6 table), use option 1 “Phenotype highly specific for gene.” Confirmed *de novo* with confirmed maternity worth 2.0 points per proband. Assumed *de novo* with assumed maternity worth 1.0 points per proband. Total 1.0 points to use at the moderate strength.

Modification Type: Gene-specific

Supporting

Use SVI point scale for counting cases (see Table 1 on PS2_PM6 table), use option 1 “Phenotype highly specific for gene.” Confirmed *de novo* with confirmed maternity worth 2.0 points per proband. Assumed *de novo* with assumed maternity worth 1.0 points per proband. Total 0.5 points to use at the supporting strength.

Modification Type: Gene-specific

PS3

Original ACMG Summary

Well-established in vitro or in vivo functional studies supportive of a damaging effect on the gene or gene product.

Note: Functional studies that have been validated and shown to be reproducible and robust in a clinical diagnostic laboratory setting are considered the most well-established.

VCEP Specifications: See RS1 Functional Assay sheet.

Strong

Variants tested in high quality knock-out/knock-in variant mouse lines can meet PS3 at the strong level.

Modification Type: Gene-specific,Strength

Supporting

Use at the supporting strength for most assays (see Functional Assays Table).

Modification Type: Gene-specific,Strength

PS4

Original ACMG Summary

The prevalence of the variant in affected individuals is significantly increased compared to the prevalence in controls.
Note 1: Relative risk (RR) or odds ratio (OR), as obtained from case-control studies, is >5.0 and the confidence interval around the estimate of RR or OR does not include 1.0. See manuscript for detailed guidance.
Note 2: In instances of very rare variants where case-control studies may not reach statistical significance, the prior observation of the variant in multiple unrelated patients with the same phenotype, and its absence in controls, may be used as moderate level of evidence.

VCEP Specifications: **Note that probands being considered for any pathogenic phenotype codes (e.g. PP1, PP4, PM6, PS2, PS4) at any strength must have the following phenotype characteristics: affected males should have some functional vision impairment by age 13, with either observed foveo-macular changes or ERG measurement of a subnormal B wave.**

Very Strong

Use of this code requires 9 or more probands, each with retinoschisis.
PM2_Supporting must be met.

Modification Type: Gene-specific,Strength

Strong

Use of this code requires 5-8 probands, each with retinoschisis. PM2_Supporting must be met.

Modification Type: Gene-specific,Strength

Moderate

Use of this code at the moderate strength requires 3-4 probands diagnosed with retinoschisis. PM2_Supporting must be met.

Modification Type: Gene-specific,Strength

Supporting

Use of this code at the supporting strength requires 1 proband diagnosed with retinoschisis, and a second proband answering the PP4 requirements. PM2_Supporting must be met.

Or 2 probands, each with retinoschisis. PM2_Supporting must be met.

Modification Type: Gene-specific,Strength

PM1

Original ACMG Summary

Located in a mutational hot spot and/or critical and well-established functional domain (e.g. active site of an enzyme) without benign variation.

Strong

Applies to variants in amino acids p.Cys40, p.Cys59, p.Cys63, p.Cys110, p.Cys142, p.Cys219, and p.Cys223 which form disulfide bridges required for structure of the retinoschisin monomer, dimers, or octamers.

Modification Type: Gene-specific

Moderate

Applies to amino acids p.Glu72, p.Trp122, p.Trp163, p.Arg200, p.Glu215 which form salt bridges that are required for higher order structure of octamers and paired octamers.

Modification Type: Gene-specific

PM2

Original ACMG Summary

Absent from controls (or at extremely low frequency if recessive) in Exome Sequencing Project, 1000 Genomes or Exome Aggregation Consortium.
Caveat: Population data for indels may be poorly called by next generation sequencing.

Supporting

At low frequency in males in population databases. Use <$2.0x10^{-6}$ for cut off. This is defined relative to the BA1 cutoff.

Modification Type: Clarification,Gene-specific

PM3

Original ACMG Summary

For recessive disorders, detected in trans with a pathogenic variant
Note: This requires testing of parents (or offspring) to determine phase.

Not Applicable

PM4

Original ACMG Summary

Protein length changes due to in-frame deletions/insertions in a non-repeat region or stop-loss variants.

VCEP Specifications: Use SVI specification.

Moderate

In-frame deletion/insertions smaller than one whole exon, in a non-repetitive region, or stop-loss variants. Variant must not be considered in any PVS1 criteria. Variant must also meet PM2.

Modification Type: General recommendation,Gene-specific

PM5

Original ACMG Summary

Novel missense change at an amino acid residue where a different missense change

determined to be pathogenic has been seen before.
Example: Arg156His is pathogenic; now you observe Arg156Cys.
Caveat: Beware of changes that impact splicing rather than at the amino acid/protein level.

Moderate

Same residue as a previously established pathogenic variant classified using these specifications (assessed independently of PM5) .

The novel change must not affect splicing (SpliceAI ≤ 0.2), must meet PP3, and have a Grantham score equal or greater than the previously published variants.

Modification Type: Clarification,Gene-specific

Supporting

Same residue as a previously established likely pathogenic variant classified using these specifications (assessed independently of PM5) .

The novel change must not affect splicing (SpliceAI ≤ 0.2), must meet PP3, and have a Grantham score equal or greater than the previously published variants.

Modification Type: Gene-specific

PM6

Original ACMG Summary

Assumed de novo, but without confirmation of paternity and maternity.

Not Applicable

Comments: See PS2 for de novo data.

PP1

Original ACMG Summary

Co-segregation with disease in multiple affected family members in a gene definitively known to cause the disease.
Note: May be used as stronger evidence with increasing segregation data.

VCEP Specifications **Note that probands being considered for any pathogenic phenotype codes (e.g. PP1, PP4, PM6, PS2, PS4) at any strength must have the following phenotype characteristics: affected males should have some functional vision impairment by age 13,**

with either observed foveo-macular changes or ERG measurement of a subnormal B wave.

Strong

Use of this code requires ≥3 meioses in 1 or 2 families.

Only phenotype positive male relatives with the same variant identified in the proband should be counted as segregations.

Modification Type: Gene-specific,Strength

Moderate

2 meioses in a family (e.g. 2 brothers and mother genotyped; 3 brothers without mother's genotype; or uncle and nephew)

Only phenotype positive male relatives with the same variant identified in the proband should be counted as segregations.

Modification Type: Gene-specific,Strength

Supporting

1 meiosis in a family

Only phenotype positive male relatives with the same variant identified in the proband should be counted as segregations.

Modification Type: Gene-specific,Strength

PP2

Original ACMG Summary

Missense variant in a gene that has a low rate of benign missense variation and where missense variants are a common mechanism of disease.

Not Applicable

Comments: Loss of function variants are underrepresented. The Z score in GnomAD is 0.97. The pLoF eligible alleles for this code is 0/303.

PP3

Original ACMG Summary

Multiple lines of computational evidence support a deleterious effect on the gene or gene product (conservation, evolutionary, splicing impact, etc.).

Caveat: As many in silico algorithms use the same or very similar input for their predictions, each algorithm should not be counted as an independent criterion. PP3 can be used only once in any evaluation of a variant.

VCEP Specifications: Use the SpliceAI flowchart included in the RS1-specific PVS1 Decision Tree file.

Strong

Applies to missense variants with a REVEL score above 0.931, that are not predicted to disrupt splicing (with a SpliceAI score less than 0.2). This criteria is met at the strong level.

Modification Type: General recommendation

Moderate

Applies to missense variants with a REVEL score between 0.773 and 0.931, that are not predicted to disrupt splicing (with a SpliceAI score less than 0.2). This criteria is met at the moderate level.

Modification Type: General recommendation

Supporting

Applies to missense variants with a REVEL score between 0.644 and 0.772 that are not predicted to disrupt splicing (with a SpliceAI score less than 0.2).

Modification Type: General recommendation

PP4

Original ACMG Summary

Patient's phenotype or family history is highly specific for a disease with a single genetic etiology.

VCEP Specifications: **Note that probands being considered for any pathogenic phenotype codes (e.g. PP1, PP4, PM6, PS2, PS4) at any strength must have the following phenotype characteristics: affected males should have some functional vision impairment by age 13, with a diagnosis of Retinoschisis and an image of schisis.**

Moderate

A male proband diagnosed with retinoschisis by the age of 13 with visual acuity

impairment, showing schisis, and having retinal detachment.

Modification Type: Clarification,Gene-specific,Strength

Supporting

A male proband diagnosed with retinoschisis by the age of 13 with visual acuity impairment and showing schisis.

Modification Type: Clarification,Gene-specific,Strength

PP5

Original ACMG Summary

Reputable source recently reports variant as pathogenic, but the evidence is not available to the laboratory to perform an independent evaluation.

Not Applicable

This criterion is not for use as recommended by the ClinGen Sequence Variant Interpretation VCEP Review Committee. PubMed : 29543229

BA1

Original ACMG Summary

Allele frequency is above 5% in Exome Sequencing Project, 1000 Genomes or Exome Aggregation Consortium.

VCEP Specifications: Any variant meeting both BA1 and PP3 must be reviewed by the VCEP.

Stand Alone

Allele frequency ≥ $2x10^{-4}$ in males in population databases (Exome Sequencing Project, 1000 Genomes or Exome Aggregation Consortium) in the subpopulation with the highest frequency.

Modification Type: Clarification

BS1

Original ACMG Summary

Allele frequency is greater than expected for disorder.

VCEP Specifications: Variants meeting both BS1 and PP3 must be reviewed by the VCEP.

Strong

Allele frequency ≥ $2x10^{-5}$ in males in population databases.
- The highest allele frequency in population databases should be used

Modification Type: Gene-specific

BS2

Original ACMG Summary

Observed in a healthy adult individual for a recessive (homozygous), dominant (heterozygous), or X-linked (hemizygous) disorder, with full penetrance expected at an early age.

Strong

Only count males over age 30 with a documented eye exam without retinoschisis.

BS2_strong for variants observed in at least 3 unaffected males.

Modification Type: Disease-specific,Gene-specific

BS3

Original ACMG Summary

Well-established in vitro or in vivo functional studies show no damaging effect on protein function or splicing.

Not Applicable

Comments: The many secretion assays have not tested enough benign variants to meet the threshold in PMID 31892348 Supplemental Table 1 or Supplemental Table 2.

BS4

Original ACMG Summary

Lack of segregation in affected members of a family.
Caveat: The presence of phenocopies for common phenotypes (i.e. cancer, epilepsy) can mimic lack of segregation among affected individuals. Also, families may have more than

one pathogenic variant contributing to an autosomal dominant disorder, further confounding an apparent lack of segregation.

Supporting

Paternal inheritance is inconsistent with this gene. For RS1, unaffected males over age 10 who have been examined could be used to establish this code if they have no schisis and good acuity.

Modification Type: Gene-specific

BP1

Original ACMG Summary

Missense variant in a gene for which primarily truncating variants are known to cause disease.

Not Applicable

BP2

Original ACMG Summary

Observed in trans with a pathogenic variant for a fully penetrant dominant gene/disorder or observed in cis with a pathogenic variant in any inheritance pattern.

Not Applicable

Comments: For this X-linked gene, only variants in cis with a pathogenic variant in RS1 in an affected male could be used, but these variants could have combined effect. The VCEP does not use this code.

BP3

Original ACMG Summary

In frame-deletions/insertions in a repetitive region without a known function.

Not Applicable

BP4

Original ACMG Summary

Multiple lines of computational evidence suggest no impact on gene or gene product

(conservation, evolutionary, splicing impact, etc)
Caveat: As many in silico algorithms use the same or very similar input for their predictions, each algorithm cannot be counted as an independent criterion. BP4 can be used only once in any evaluation of a variant.

Strong

Stand Alone criteria applies to:

Missense variants: REVEL ≤ 0.003 with SpliceAI ≤ 0.1

Strong criteria applies to:

Missense variants: REVEL between 0.004 and 0.016 with SpliceAI ≤ 0.1

Modification Type: General recommendation

Moderate

Applies to:

Missense variants: REVEL between 0.017 and 0.183 with SpliceAI ≤ 0.1

Modification Type: General recommendation

Supporting

Applies to:

Missense variants: REVEL between 0.184 and 0.290 with SpliceAI ≤ 0.1

or

Synonymous variants or noncoding variants: SpliceAI ≤ 0.1

Modification Type: General recommendation

BP5

Original ACMG Summary

Variant found in a case with an alternate molecular basis for disease.

Not Applicable

BP6

Original ACMG Summary

Reputable source recently reports variant as benign, but the evidence is not available to

the laboratory to perform an independent evaluation.

Not Applicable

This criterion is not for use as recommended by the ClinGen Sequence Variant Interpretation VCEP Review Committee. PubMed : 29543229

BP7

Original ACMG Summary

A synonymous variant for which splicing prediction algorithms predict no impact to the splice consensus sequence nor the creation of a new splice site AND the nucleotide is not highly conserved.

VCEP Specifications: This code may be used with BP4 as described in the RS1-specific PVS1 Decision Tree file splicing document and Walker, et al, 2023.

Strong

Applies to splicing assay data demonstrating a variant is not associated with aberrantly spliced transcript(s) relative to transcript profiles in controls. See RS1 PVS1 Decision Tree. As per Walker et al, 2023 (PMID 37352859) guidance.

Modification Type: General recommendation,Gene-specific

Supporting

Applies to:

Synonymous variants or noncoding variants with no impact on splicing (SpliceAI ≤ 0.1)

AND

PhyloP < 0.1 for conservation

Modification Type: Clarification,Gene-specific

Rules for Combining Criteria

Pathogenic

1 Very Strong *(PVS1, PS2_Very Strong, PS4_Very Strong)* **AND ≥ 1 Strong** *(PVS1_Strong, PS1, PS2, PS3, PS4, PM1_Strong, PP1_Strong, PP3_Strong)*

1 Very Strong *(PVS1, PS2_Very Strong, PS4_Very Strong)* **AND ≥ 2 Moderate** *(PVS1_Moderate, PS1_Moderate, PS2_Moderate, PS4_Moderate, PM1, PM4, PM5, PP1_Moderate, PP3_Moderate, PP4_Moderate)*

1 Very Strong *(PVS1, PS2_Very Strong, PS4_Very Strong)* **AND 1 Moderate** *(PVS1_Moderate, PS1_Moderate, PS2_Moderate, PS4_Moderate, PM1, PM4, PM5, PP1_Moderate, PP3_Moderate, PP4_Moderate)* **AND 1 Supporting** *(PS1_Supporting, PS2_Supporting, PS3_Supporting, PS4_Supporting, PM2_Supporting, PM5_Supporting, PP1, PP3, PP4)*

1 Very Strong *(PVS1, PS2_Very Strong, PS4_Very Strong)* **AND ≥ 2 Supporting** *(PS1_Supporting, PS2_Supporting, PS3_Supporting, PS4_Supporting, PM2_Supporting, PM5_Supporting, PP1, PP3, PP4)*

≥ 2 Strong *(PVS1_Strong, PS1, PS2, PS3, PS4, PM1_Strong, PP1_Strong, PP3_Strong)*

1 Strong *(PVS1_Strong, PS1, PS2, PS3, PS4, PM1_Strong, PP1_Strong, PP3_Strong)* **AND ≥ 3 Moderate** *(PVS1_Moderate, PS1_Moderate, PS2_Moderate, PS4_Moderate, PM1, PM4, PM5, PP1_Moderate, PP3_Moderate, PP4_Moderate)*

1 Strong *(PVS1_Strong, PS1, PS2, PS3, PS4, PM1_Strong, PP1_Strong, PP3_Strong)* **AND 2 Moderate** *(PVS1_Moderate, PS1_Moderate, PS2_Moderate, PS4_Moderate, PM1, PM4, PM5, PP1_Moderate, PP3_Moderate, PP4_Moderate)* **AND ≥ 2 Supporting** *(PS1_Supporting, PS2_Supporting, PS3_Supporting, PS4_Supporting, PM2_Supporting, PM5_Supporting, PP1, PP3, PP4)*

1 Strong *(PVS1_Strong, PS1, PS2, PS3, PS4, PM1_Strong, PP1_Strong, PP3_Strong)* **AND 1 Moderate** *(PVS1_Moderate, PS1_Moderate, PS2_Moderate, PS4_Moderate, PM1, PM4, PM5, PP1_Moderate, PP3_Moderate, PP4_Moderate)* **AND ≥ 4 Supporting** *(PS1_Supporting, PS2_Supporting, PS3_Supporting, PS4_Supporting, PM2_Supporting, PM5_Supporting, PP1, PP3, PP4)*

Likely Pathogenic

1 Very Strong *(PVS1, PS2_Very Strong, PS4_Very Strong)* **AND 1 Moderate** *(PVS1_Moderate, PS1_Moderate, PS2_Moderate, PS4_Moderate, PM1, PM4, PM5, PP1_Moderate, PP3_Moderate, PP4_Moderate)*

1 Strong *(PVS1_Strong, PS1, PS2, PS3, PS4, PM1_Strong, PP1_Strong, PP3_Strong)* **AND 1 Moderate** *(PVS1_Moderate, PS1_Moderate, PS2_Moderate, PS4_Moderate, PM1, PM4, PM5, PP1_Moderate, PP3_Moderate, PP4_Moderate)*

1 Strong *(PVS1_Strong, PS1, PS2, PS3, PS4, PM1_Strong, PP1_Strong, PP3_Strong)* **AND ≥ 2 Supporting** *(PS1_Supporting, PS2_Supporting, PS3_Supporting, PS4_Supporting, PM2_Supporting, PM5_Supporting, PP1, PP3, PP4)*

≥ 3 Moderate *(PVS1_Moderate, PS1_Moderate, PS2_Moderate, PS4_Moderate, PM1, PM4, PM5, PP1_Moderate, PP3_Moderate, PP4_Moderate)*

2 Moderate *(PVS1_Moderate, PS1_Moderate, PS2_Moderate, PS4_Moderate, PM1, PM4, PM5, PP1_Moderate, PP3_Moderate, PP4_Moderate)* **AND ≥ 2 Supporting** *(PS1_Supporting, PS2_Supporting, PS3_Supporting, PS4_Supporting, PM2_Supporting, PM5_Supporting, PP1, PP3, PP4)*

1 Moderate *(PVS1_Moderate, PS1_Moderate, PS2_Moderate, PS4_Moderate, PM1, PM4, PM5, PP1_Moderate, PP3_Moderate, PP4_Moderate)* **AND ≥ 4 Supporting** *(PS1_Supporting, PS2_Supporting, PS3_Supporting, PS4_Supporting, PM2_Supporting, PM5_Supporting, PP1, PP3, PP4)*

1 Strong *(PVS1_Strong, PS1, PS2, PS3, PS4, PM1_Strong, PP1_Strong, PP3_Strong)* **AND 2 Moderate** *(PVS1_Moderate, PS1_Moderate, PS2_Moderate, PS4_Moderate, PM1, PM4, PM5, PP1_Moderate, PP3_Moderate, PP4_Moderate)*

Benign

≥ 2 Strong *(BS1, BS2, BP4_Strong)*

1 Stand Alone *(BA1)*

Likely Benign

1 Strong *(BS1, BS2, BP4_Strong)* **AND 1 Supporting** *(BS4_Supporting, BP4, BP7)*

≥ 2 Supporting *(BS4_Supporting, BP4, BP7)*

Files & Images

RS1 PS2/PM6 Tables: OTC data for a proband showing schisis increases the available points.

RS1 PVS1 Decision Tree: PDF file of PVS1 Decision Tree for RS1 with splicing information.

RS1 Functional Evidence Assays for PS3 / BS3: Excel file of published functional assays, publications , and if they have been approved for use by the VCEP. Check the Approved row before use of any publication for a particular assay. 01/01/2025

References

1. Heymann JB Vijayasarathy C et al. *Advances in understanding the molecular structure of retinoschisin while questions remain of biological function.* **Prog Retin Eye Res** (2023) 95 p. 101147. 10.1016/j.preteyeres.2022.101147 36402656
2. Wang T Waters CT et al. *Intracellular retention of mutant retinoschisin is the pathological mechanism underlying X-linked retinoschisis.* **Hum Mol Genet** (2002) 11 (24) p. 3097-105. 10.1093/hmg/11.24.3097 12417531
3. Wu WW Molday RS *Defective discoidin domain structure, subunit assembly, and endoplasmic reticulum processing of retinoschisin are primary mechanisms responsible for X-linked retinoschisis.* **J Biol Chem** (2003) 278 (30) p. 28139-46. 10.1074/jbc.M302464200 12746437
4. Wang T Zhou A et al. *Molecular pathology of X linked retinoschisis: mutations interfere with retinoschisin secretion and oligomerisation.* **Br J Ophthalmol** (2006) 90 (1) p. 81-6. 10.1136/bjo.2005.078048 16361673
5. Dyka FM Molday RS *Coexpression and interaction of wild-type and missense RS1 mutants associated with X-linked retinoschisis: its relevance to gene therapy.* **Invest Ophthalmol Vis Sci** (2007) 48 (6) p. 2491-7. 10.1167/iovs.06-1465 17525175
6. Gleghorn LJ Trump D et al. *Wild-type and missense mutants of retinoschisin co-assemble resulting in either intracellular retention or incorrect assembly of the functionally active octamer.* **Biochem J** (2009) 425 (1) p. 275-83. 10.1042/BJ20091179 19849666
7. Vijayasarathy C Sui R et al. *Molecular mechanisms leading to null-protein product from retinoschisin (RS1) signal-sequence mutants in X-linked retinoschisis (XLRS) disease.* **Hum Mutat** (2010) 31 (11) p. 1251-60. 10.1002/humu.21350 20809529
8. Chen D Xu T et al. *Recapitulating X-Linked Juvenile Retinoschisis in Mouse Model by Knock-In Patient-Specific Novel Mutation.* **Front Mol Neurosci** (2017) 10 p. 453. 10.3389/fnmol.2017.00453 29379415
9. Sudha D Neriyanuri S et al. *Understanding variable disease severity in X-linked retinoschisis: Does RS1 secretory mechanism determine disease severity?* **PLoS One** (2018) 13 (5) p. e0198086. 10.1371/journal.pone.0198086 29851975
10. Plössl K Schmid V et al. *Pathomechanism of mutated and secreted retinoschisin in X-linked juvenile retinoschisis.* **Exp Eye Res** (2018) 177 p. 23-34. 10.1016/j.exer.2018.07.021 30040949
11. Heymann JB Vijayasarathy C et al. *Cryo-EM of retinoschisin branched networks suggests an intercellular adhesive scaffold in the retina.* **J Cell Biol** (2019) 218 (3) p. 1027-1038. 10.1083/jcb.201806148 30630865
12. Liu Y Kinoshita J et al. *Mouse models of X-linked juvenile retinoschisis have an early onset phenotype, the severity of which varies with genotype.* **Hum Mol Genet** (2019) 28 (18) p. 3072-3090. 10.1093/hmg/ddz122 31174210
13. Bender C Woo EG et al. *Predominant Founder Effect among Recurrent Pathogenic Variants for an X-Linked Disorder.* **Genes (Basel)** (2022) 13 (4) 10.3390/genes13040675 35456481
14. Tavtigian SV Harrison SM et al. *Fitting a naturally scaled point system to the ACMG/AMP variant classification guidelines.* **Hum Mutat** (2020) 41 (10) p. 1734-1737. 10.1002/humu.24088 32720330

15. Abou Tayoun AN Pesaran T et al. *Recommendations for interpreting the loss of function PVS1 ACMG/AMP variant criterion.* **Hum Mutat** (2018) 39 (11) p. 1517-1524. 10.1002/humu.23626 30192042

16. Walker LC Hoya M et al. *Using the ACMG/AMP framework to capture evidence related to predicted and observed impact on splicing: Recommendations from the ClinGen SVI Splicing Subgroup.* **Am J Hum Genet** (2023) 110 (7) p. 1046-1067. 10.1016/j.ajhg.2023.06.002 37352859
