# Supplementary material for: Development of RS1-specific ACMG/AMP variant classification criteria with pilot variant curation
Source: Hum Genet. 2026 Sep 4;145(1):75. doi: 10.1007/s00439-026-02871-0 (PMC13545035; doi:10.1007/s00439-026-02871-0)
Supplement: Supplementary file 2 [file 439_2026_2871_MOESM4_ESM.pdf]

Evaluation Summary

NM_000330.4(RS1):c.7C>G (p.Arg3Gly)

ClinVar VariationID: 757382

ClinGen Allele Registry ID: CA10360837

Interpretation owner: X-linked Inherited Retinal Disease

Auto-calculated Classification: Benign

Modified Classification: None

Reason for modified classification: None

Interpretation Status: PROVISIONAL

Date interpretation saved: 2024 Oct 04, 2:48 pm

Disease: X-linked retinoschisis

Mode of Inheritance: X-linked inheritance

Specification Document: None

Evidence Summary

The NM_000330.4(RS1):c.7C>G variant is a missense variant encoding the substitution of Arginine with Glycine at position 3. This variant is present in gnomAD v.4.1.0 at a frequency of 0.001335 among hemizygous individuals, with 528 variant alleles / 395462 total alleles, which is higher than the ClinGen X-linked IRD VCEP BA1 threshold of >0.0002 (BA1). The computational predictor REVEL gives a score of 0.203, which is below the ClinGen X-linked IRD VCEP threshold of <0.291 and predicts a non-damaging effect on the RS1 function. Additionally, the splicing impact predictor SpliceAI gives a delta score of 0.01, which is below the ClinGen X-linked IRD VCEP recommended threshold of <0.1 and does not strongly predict an impact on splicing (BP4). The variant has been reported to segregate with retinal dystrophy through 1 meiosis in a family- a mother and an affected son. The mother is unaffected- the PP1 code could not be applied. (PMID: 38317323). This variant has been reported in at least 1 proband meeting the PS4 requirement of a male diagnosed with retinoschisis (PMIDs: 38317323). However, PS4_Supporting requires at least 2 unrelated probands, so this criterion was not met. In summary, this variant is classified as benign for X-linked retinoschisis based on the ACMG/AMP criteria applied, as specified by the ClinGen X-linked IRD VCEP: BA1, and BP4. (VCEP specifications version PILOT; date of approval 11/2/2024).

Criteria meeting an evaluation strength

B/P	Criteria	Criteria Descriptions	Modified	Status	Explanation
✔	BA1	Allele frequency greater than 5% in a population database	No	met	This variant is present in gnomAD v.4.1.0 at a frequency of 0.001335 among hemizygous individuals, with 528 variant alleles / 395462 total alleles, which is higher than the ClinGen X-linked IRD VCEP BA1 threshold of >0.0002 (BA1).
✔	BP4	Multiple lines of computational evidence suggest no impact on gene /gene product	No	met	The computational predictor REVEL gives a score of 0.203, which is below the ClinGen X-linked IRD VCEP threshold of <0.291 and predicts a non-damaging effect on the RS1 function. Additionally, the splicing impact predictor SpliceAI gives a delta score of 0.01, which is below the ClinGen X-linked IRD VCEP recommended threshold of <0.1 and does not strongly predict an impact on splicing (BP4).

Criteria evaluated as 'Not met'

B/P	Criteria	Criteria Descriptions	Modified	Status	Explanation
✖	PS4	Prevalence in affecteds statistically increased over controls	N/A	Not Met	This variant has been reported in at least 1 proband meeting the PS4 requirement of a male diagnosed with retinoschisis (PMIDs: 38317323). However, PS4_Supporting requires at least 2 unrelated probands, so this criterion was not met.
✖	PP1	Cosegregation with disease in multiple affected family members	N/A	Not Met	The variant has been reported to segregate with retinal dystrophy through 1 meiosis in a family- a mother and an affected son. The mother is unaffected- the PP1 code could not be applied. (PMID: 38317323).

Criteria 'Not yet evaluated'

B/P	Criteria	Criteria Descriptions	Modified	Status	Explanation
○	PVS1	Predicted null variant in a gene where LOF is a known mechanism of disease	N/A	Not Evaluated	
○	BS1	MAF is too high for disorder	N/A	Not Evaluated	

B/P	Criteria	Criteria Descriptions	Modified	Status	Explanation
	BS2	Observation in controls inconsistent with disease penetrance	N/A	Not Evaluated	
	BS3	Well-established functional studies show no deleterious effect	N/A	Not Evaluated	
	BS4	Non-segregation with disease	N/A	Not Evaluated	
	PS1	Same amino acid change as an established pathogenic variant	N/A	Not Evaluated	
	PS2	De novo (paternity and maternity confirmed)	N/A	Not Evaluated	
	PS3	Well-established functional studies show a deleterious effect	N/A	Not Evaluated	
	PM1	Mutational hot spot or well-studied functional domain without benign variation	N/A	Not Evaluated	
	PM2	Absent in population databases	N/A	Not Evaluated	
	PM3	For recessive disorders, detected in trans with a pathogenic variant	N/A	Not Evaluated	
	PM4	Protein length changing variant	N/A	Not Evaluated	

B/P	Criteria	Criteria Descriptions	Modified	Status	Explanation
○	PM5	Novel missense change at an amino acid residue where a different pathogenic missense change has been seen before	N/A	Not Evaluated	
○	PM6	De novo (without paternity & maternity confirmed)	N/A	Not Evaluated	
○	BP1	Missense in gene where primarily truncating cause disease	N/A	Not Evaluated	
○	BP2	Observed in trans with path. variant for dominant disorder or cis any inheritance	N/A	Not Evaluated	
○	BP3	In-frame indels in repeat w/out known function	N/A	Not Evaluated	
○	BP5	Found in case with an alternate cause	N/A	Not Evaluated	
○	BP6	Reputable source w/out shared data = benign	N/A	Not Evaluated	
○	BP7	Silent variant predicted with no splice impact	N/A	Not Evaluated	
○	PP2	Missense in gene with low rate of benign missense variants and path. missenses common	N/A	Not Evaluated	
○	PP3	Multiple lines of computational evidence support a deleterious effect on the gene /gene product	N/A	Not Evaluated	

B/P	Criteria	Criteria Descriptions	Modified	Status	Explanation
	PP4	Patient's phenotype or FH highly specific for gene	N/A	Not Evaluated	
	PP5	Reputable source = pathogenic	N/A	Not Evaluated	

Print PDF

Evaluation Summary

NM_000330.4(RS1):c.35T>A (p.Leu12His)

ClinVar VariationID: 98946

ClinGen Allele Registry ID: CA226701

Interpretation owner: X-linked Inherited Retinal Disease

Auto-calculated Classification: Uncertain significance - insufficient evidence

Modified Classification: None

Reason for modified classification: None

Interpretation Status: PROVISIONAL

Date interpretation saved: 2024 Oct 04, 3:09 pm

Disease: X-linked retinoschisis

Mode of Inheritance: X-linked inheritance (recessive)

Specification Document: None

Evidence Summary

The NM_000330.4(RS1):c.35T>A variant is a missense variant encoding the substitution of Leucine with Histidine at position 12. This variant is absent from hemizygous individuals in gnomAD v4.1.0 (PM2_Supporting). COS cells exogenously expressing the variant exhibit loss of RS1 secretion into the medium relative to the wild-type control (PMID: 12417531, PS3_Supporting). The computational predictor REVEL gives a score of 0.633, which is between the ClinGen X-linked IRD VCEP threshold of 0.664 to 0.290 and does not predict a damaging effect on RS1 function. Additionally, the splicing impact predictor SpliceAI gives a score of 0.00, which is below the ClinGen X-linked IRD VCEP recommended threshold of ≥0.2 and does not strongly predict an impact on splicing. Collectively, both BP4 and PP3 codes do not apply. This variant has been reported in at least 2 apparently unrelated probands meeting the PS4 requirements of a male diagnosed with XLRS (PMIDs: 9618178, 34822951, 31848469, 32860923, 28348004, 35456481, PS4_supporting). In summary, this variant is classified as a variant of uncertain significance for X-linked retinoschisis based on the ACMG/AMP criteria applied, as specified by the ClinGen X-linked IRD VCEP: PS3_supporting, PS4_supporting, and PM2_supporting. (VCEP specifications version PILOT; date of approval 6/13/2024).

Criteria meeting an evaluation strength

B/P	Criteria	Criteria Descriptions	Modified	Status	Explanation
✔	PM2	Absent in population databases	Yes	PM2_supporting	This variant is absent from hemizygous individuals in gnomAD v4.1.0 (PM2_Supporting).
✔	PS3	Well-established functional studies show a deleterious effect	Yes	PS3_supporting	COS cells exogenously expressing the variant exhibit loss of RS1 secretion into the medium relative to the wild-type control (PMID: 12417531, PS3_Supporting).
✔	PS4	Prevalence in affecteds statistically increased over controls	Yes	PS4_supporting	This variant has been reported in at least 2 apparently unrelated probands meeting the PS4 requirements of a male diagnosed with XLRS (PMIDs: PMIDs: 9618178, 34822951, 31848469, 32860923, 28348004, 35456481, PS4_supporting)

Criteria evaluated as 'Not met'

B/P	Criteria	Criteria Descriptions	Modified	Status	Explanation
✖	BP4	Multiple lines of computational evidence suggest no impact on gene /gene product	N/A	Not Met	The computational predictor REVEL gives a score of 0.633, which is between the ClinGen X-linked IRD VCEP threshold of 0.664 to 0.290 and does not predict a damaging effect on RS1 function. Additionally, the splicing impact predictor SpliceAI gives a score of 0.00, which is below the ClinGen X-linked IRD VCEP recommended threshold of ≥0.2 and does not strongly predict an impact on splicing. Collectively, both BP4 and PP3 codes do not apply.
✖	PP3	Multiple lines of computational evidence support a deleterious effect on the gene /gene product	N/A	Not Met	The computational predictor REVEL gives a score of 0.633, which is between the ClinGen X-linked IRD VCEP threshold of 0.664 to 0.290 and does not predict a damaging effect on RS1 function. Additionally, the splicing impact predictor SpliceAI gives a score of 0.00, which is below the ClinGen X-linked IRD VCEP recommended threshold of ≥0.2 and does not strongly predict an impact on splicing. Collectively, both BP4 and PP3 codes do not apply.

Criteria 'Not yet evaluated'

B/P	Criteria	Criteria Descriptions	Modified	Status	Explanation
○	BA1	Allele frequency greater than 5% in a population database	N/A	Not Evaluated	
○	PVS1	Predicted null variant in a gene where LOF is a known mechanism of disease	N/A	Not Evaluated	
○	BS1	MAF is too high for disorder	N/A	Not Evaluated	
○	BS2	Observation in controls inconsistent with disease penetrance	N/A	Not Evaluated	
○	BS3	Well-established functional studies show no deleterious effect	N/A	Not Evaluated	
○	BS4	Non-segregation with disease	N/A	Not Evaluated	
○	PS1	Same amino acid change as an established pathogenic variant	N/A	Not Evaluated	
○	PS2	De novo (paternity and maternity confirmed)	N/A	Not Evaluated	
○	PM1	Mutational hot spot or well-studied functional domain without benign variation	N/A	Not Evaluated	

B/P	Criteria	Criteria Descriptions	Modified	Status	Explanation
○	PM3	For recessive disorders, detected in trans with a pathogenic variant	N/A	Not Evaluated	
○	PM4	Protein length changing variant	N/A	Not Evaluated	
○	PM5	Novel missense change at an amino acid residue where a different pathogenic missense change has been seen before	N/A	Not Evaluated	
○	PM6	De novo (without paternity & maternity confirmed)	N/A	Not Evaluated	
○	BP1	Missense in gene where primarily truncating cause disease	N/A	Not Evaluated	
○	BP2	Observed in trans with path. variant for dominant disorder or cis any inheritance	N/A	Not Evaluated	
○	BP3	In-frame indels in repeat w/out known function	N/A	Not Evaluated	
○	BP5	Found in case with an alternate cause	N/A	Not Evaluated	
○	BP6	Reputable source w/out shared data = benign	N/A	Not Evaluated	
○	BP7	Silent variant predicted with no splice impact	N/A	Not Evaluated	

B/P	Criteria	Criteria Descriptions	Modified	Status	Explanation
○	PP1	Cosegregation with disease in multiple affected family members	N/A	Not Evaluated	
○	PP2	Missense in gene with low rate of benign missense variants and path. missenses common	N/A	Not Evaluated	
○	PP4	Patient's phenotype or FH highly specific for gene	N/A	Not Evaluated	
○	PP5	Reputable source = pathogenic	N/A	Not Evaluated	

Print PDF

Evaluation Summary

NM_000330.4(RS1):c.78+5G>A

ClinGen Allele Registry ID: CA2695231369
Interpretation owner: X-linked Inherited Retinal Disease
Auto-calculated Classification: Likely pathogenic
Modified Classification: None
Reason for modified classification: None

Interpretation Status: PROVISIONAL
Date interpretation saved: 2024 Oct 07, 10:38 am
Disease: X-linked retinoschisis
Mode of Inheritance: X-linked inheritance (recessive)
Specification Document: None

Evidence Summary

The NM_000330.4(RS1):c.78+5G>A variant is an intronic variant at intron 2, located 5 nucleotides after exon 2. This variant is absent from hemizygous individuals in gnomAD v4.1.0 (PM2_Supporting). The splicing impact predictor SpliceAI gives a score of 0.64 (Donor Loss), which is above the ClinGen X-linked IRD VCEP recommended threshold of ≥0.2 and predicts a damaging impact on splicing (PP3). This variant is outside the +/- 1,2 dinucleotide and meets the PP3 code due to predicted splicing disruption and another predicted splicing variant, NM_000330.4(RS1):c.78+1G>A, was previously classified as a pathogenic variant in the same donor motif for the RPGR-related X-linked IRD VCEP (PS1_moderate). The variant has been reported to segregate with retinal dystrophy through 1 meiosis in two families . (PMIDs: 19390641, 34645606). Two unrelated families with a carrier mother and an affected son, were reported however, the mothers are asymptomatic and therefore not meeting the PP1 code. At least one proband harboring this variant exhibits a phenotype including X-linked inheritance (Mother was also tested genetically), diagnosed with XLRS before the age of 13 years (required), Decreased central vision acuity, and absence of retinoschisis disease in carrier females, which together are specific for X-linked retinoschisis (PMID: 19390641, this proband was counted as a PS4 code). This variant has been reported in at least 3 apparently unrelated probands meeting the PS4 requirements of a male diagnosed with XLRS (PMIDs: 34645606, 35456481, 19390641, PS4_Moderate). In summary, this variant is classified as likely pathogenic for X-linked retinoschisis based on the ACMG/AMP criteria applied, as specified by the ClinGen X-linked IRD VCEP: PM2_supporting, PP3, PS1_moderate, and PS4_moderate ((VCEP specifications version PILOT; date of approval 6/13/24).

Criteria meeting an evaluation strength

B/P	Criteria	Criteria Descriptions	Modified	Status	Explanation
✓	PS1	Same amino acid change as an established pathogenic variant	Yes	PS1_moderate	This variant is outside the +/- 1,2 dinucleotide and meets the PP3 code due to predicted splicing disruption and another predicted splicing variant, NM_000330.4(RS1):c.78+1G>A, was previously classified as a pathogenic variant in the same donor motif for the RPGR-related X-linked IRD VCEP (PS1_moderate).
✓	PS4	Prevalence in affecteds statistically increased over controls	Yes	PS4_moderate	This variant has been reported in at least 3 apparently unrelated probands meeting the PS4 requirements of a male diagnosed with XLRS (PMIDs: 34645606, 35456481, 19390641, PS4_Moderate).
✓	PP3	Multiple lines of computational evidence support a deleterious effect on the gene /gene product	No	met	The splicing impact predictor SpliceAI gives a score of 0.64 (Donor Loss), which is above the ClinGen X-linked IRD VCEP recommended threshold of ≥0.2 and predicts a damaging impact on splicing (PP3).

B/P	Criteria	Criteria Descriptions	Modified	Status	Explanation
✔	PM2	Absent in population databases	Yes	PM2_supporting	This variant is absent from hemizygous individuals in gnomAD v4.1.0 (PM2_Supporting).

Criteria evaluated as 'Not met'

B/P	Criteria	Criteria Descriptions	Modified	Status	Explanation
✖	PP1	Cosegregation with disease in multiple affected family members	N/A	Not Met	The variant has been reported to segregate with retinal dystrophy through 1 meiosis in two families . (PMIDs: 19390641, 34645606). Two unrelated families with a carrier mother and an affected son, were reported however, the mothers are asymptomatic and therefore not meeting this code.

B/P	Criteria	Criteria Descriptions	Modified	Status	Explanation
✖	PP4	Patient's phenotype or FH highly specific for gene	N/A	Not Met	At least one proband harboring this variant exhibits a phenotype including X-linked inheritance (Mother was also tested genetically), diagnosed with XLRS before the age of 13 years (required), Decreased central vision acuity, and absence of retinoschisis disease in carrier females, which together are specific for X-linked retinoschisis (PMID: 19390641). This proband was counted as a PS4 code.

Criteria 'Not yet evaluated'

B/P	Criteria	Criteria Descriptions	Modified	Status	Explanation
○	BA1	Allele frequency greater than 5% in a population database	N/A	Not Evaluated	
○	PVS1	Predicted null variant in a gene where LOF is a known mechanism of disease	N/A	Not Evaluated	
○	BS1	MAF is too high for disorder	N/A	Not Evaluated	

B/P	Criteria	Criteria Descriptions	Modified	Status	Explanation
○	**BS2**	Observation in controls inconsistent with disease penetrance	N/A	Not Evaluated	
○	**BS3**	Well-established functional studies show no deleterious effect	N/A	Not Evaluated	
○	**BS4**	Non-segregation with disease	N/A	Not Evaluated	
○	**PS2**	De novo (paternity and maternity confirmed)	N/A	Not Evaluated	
○	**PS3**	Well-established functional studies show a deleterious effect	N/A	Not Evaluated	
○	**PM1**	Mutational hot spot or well-studied functional domain without benign variation	N/A	Not Evaluated	
○	**PM3**	For recessive disorders, detected in trans with a pathogenic variant	N/A	Not Evaluated	
○	**PM4**	Protein length changing variant	N/A	Not Evaluated	
○	**PM5**	Novel missense change at an amino acid residue where a different pathogenic missense change has been seen before	N/A	Not Evaluated	
○	**PM6**	De novo (without paternity & maternity confirmed)	N/A	Not Evaluated	

B/P	Criteria	Criteria Descriptions	Modified	Status	Explanation
○	BP1	Missense in gene where primarily truncating cause disease	N/A	Not Evaluated	
○	BP2	Observed in trans with path. variant for dominant disorder or cis any inheritance	N/A	Not Evaluated	
○	BP3	In-frame indels in repeat w/out known function	N/A	Not Evaluated	
○	BP4	Multiple lines of computational evidence suggest no impact on gene /gene product	N/A	Not Evaluated	
○	BP5	Found in case with an alternate cause	N/A	Not Evaluated	
○	BP6	Reputable source w/out shared data = benign	N/A	Not Evaluated	
○	BP7	Silent variant predicted with no splice impact	N/A	Not Evaluated	
○	PP2	Missense in gene with low rate of benign missense variants and path. missenses common	N/A	Not Evaluated	
○	PP5	Reputable source = pathogenic	N/A	Not Evaluated	

Print PDF

Evaluation Summary

NM_000330.4(RS1):c.295A>G (p.Asn99Asp)

ClinVar VariationID: 710170

ClinGen Allele Registry ID: CA10360704

Interpretation owner: X-linked Inherited Retinal Disease

Auto-calculated Classification: Benign

Modified Classification: None

Reason for modified classification: None

Interpretation Status: PROVISIONAL

Date interpretation saved: 2024 Oct 07, 3:52 pm

Disease: X-linked retinoschisis

Mode of Inheritance: X-linked inheritance

Specification Document: None

Evidence Summary

The NM_000330.4(RS1):c.295A>G variant is a missense variant encoding the substitution of Asparagine with Aspartic acid at position 99. This variant is present in gnomAD v4.1.0 at a frequency of 0.002748 among hemizygous individuals, with 1090 variant alleles / 396588 total alleles, which is higher than the ClinGen X-linked IRD VCEP BA1 threshold of >0.0002 (BA1). This variant has been observed in cis with the variant c.608C>T (p.Pro203Leu) (PMID: 35456481) which is classified as pathogenic by the ClinGen IRD in an individual with XLRS. The phase of the variants was confirmed by mother testing. The CSpec does not allow use of this code, the BP2 code is not met. The computational predictor REVEL gives a score of 0.443, which is between the ClinGen X-linked IRD VCEP threshold of 0.664 to 0.290 and does not predict a damaging effect on RS1 function. Additionally, the splicing impact predictor SpliceAI gives a score of 0.00, which is below the ClinGen X-linked IRD VCEP recommended threshold of ≥0.2 and does not strongly predict an impact on splicing. Collectively, both BP4 and PP3 codes do not apply. In summary, this variant is classified as benign for X-linked retinoschisis based on the ACMG/AMP criteria applied, as specified by the ClinGen X-linked IRD VCEP: BA1. VCEP specifications version PILOT; date of approval 11/02/2024.

Criteria meeting an evaluation strength

B/P	Criteria	Criteria Descriptions	Modified	Status	Explanation
✔	BA1	Allele frequency greater than 5% in a population database	No	met	This variant is present in gnomAD v4.1.0 at a frequency of 0.002748 among hemizygous individuals, with 1090 variant alleles / 396588 total alleles, which is higher than the ClinGen X-linked IRD VCEP BA1 threshold of >0.0002 (BA1).

Criteria evaluated as 'Not met'

B/P	Criteria	Criteria Descriptions	Modified	Status	Explanation
✖	BP2	Observed in trans with path. variant for dominant disorder or cis any inheritance	N/A	Not Met	This variant has been observed in cis with the variant c.608C>T (p.Pro203Leu) (PMID: 35456481) which is classified as pathogenic by the ClinGen IRD in an individual with XLRS. The phase of the variants was confirmed by mother testing. The CSpec does not allow use of this code, the BP2 code is not met.

B/P	Criteria	Criteria Descriptions	Modified	Status	Explanation
✖	BP4	Multiple lines of computational evidence suggest no impact on gene /gene product	N/A	Not Met	The computational predictor REVEL gives a score of 0.443, which is between the ClinGen X-linked IRD VCEP threshold of 0.664 to 0.290 and does not predict a damaging effect on RS1 function. Additionally, the splicing impact predictor SpliceAI gives a score of 0.00, which is below the ClinGen X-linked IRD VCEP recommended threshold of ≥0.2 and does not strongly predict an impact on splicing. Collectively, both BP4 and PP3 codes do not apply.
✖	PP3	Multiple lines of computational evidence support a deleterious effect on the gene /gene product	N/A	Not Met	The computational predictor REVEL gives a score of 0.443, which is between the ClinGen X-linked IRD VCEP threshold of 0.664 to 0.290 and does not predict a damaging effect on RS1 function. Additionally, the splicing impact predictor SpliceAI gives a score of 0.00, which is below the ClinGen X-linked IRD VCEP recommended threshold of ≥0.2 and does not strongly predict an impact on splicing. Collectively, both BP4 and PP3 codes do not apply.

Criteria 'Not yet evaluated'

B/P	Criteria	Criteria Descriptions	Modified	Status	Explanation
○	PVS1	Predicted null variant in a gene where LOF is a known mechanism of disease	N/A	Not Evaluated	
○	BS1	MAF is too high for disorder	N/A	Not Evaluated	
○	BS2	Observation in controls inconsistent with disease penetrance	N/A	Not Evaluated	
○	BS3	Well-established functional studies show no deleterious effect	N/A	Not Evaluated	
○	BS4	Non-segregation with disease	N/A	Not Evaluated	
○	PS1	Same amino acid change as an established pathogenic variant	N/A	Not Evaluated	
○	PS2	De novo (paternity and maternity confirmed)	N/A	Not Evaluated	
○	PS3	Well-established functional studies show a deleterious effect	N/A	Not Evaluated	
○	PS4	Prevalence in affecteds statistically increased over controls	N/A	Not Evaluated	
○	PM1	Mutational hot spot or well-studied functional domain without benign variation	N/A	Not Evaluated	

B/P	Criteria	Criteria Descriptions	Modified	Status	Explanation
○	PM2	Absent in population databases	N/A	Not Evaluated	
○	PM3	For recessive disorders, detected in trans with a pathogenic variant	N/A	Not Evaluated	
○	PM4	Protein length changing variant	N/A	Not Evaluated	
○	PM5	Novel missense change at an amino acid residue where a different pathogenic missense change has been seen before	N/A	Not Evaluated	
○	PM6	De novo (without paternity & maternity confirmed)	N/A	Not Evaluated	
○	BP1	Missense in gene where primarily truncating cause disease	N/A	Not Evaluated	
○	BP3	In-frame indels in repeat w/out known function	N/A	Not Evaluated	
○	BP5	Found in case with an alternate cause	N/A	Not Evaluated	
○	BP6	Reputable source w/out shared data = benign	N/A	Not Evaluated	
○	BP7	Silent variant predicted with no splice impact	N/A	Not Evaluated	

B/P	Criteria	Criteria Descriptions	Modified	Status	Explanation
	PP1	Cosegregation with disease in multiple affected family members	N/A	Not Evaluated	
	PP2	Missense in gene with low rate of benign missense variants and path. missenses common	N/A	Not Evaluated	
	PP4	Patient's phenotype or FH highly specific for gene	N/A	Not Evaluated	
	PP5	Reputable source = pathogenic	N/A	Not Evaluated	

Print PDF

Evaluation Summary

NM_000330.4(RS1):c.326+1G>A

ClinVar VariationID: 98936

ClinGen Allele Registry ID: CA226683

Interpretation owner: X-linked Inherited Retinal Disease

Auto-calculated Classification: Pathogenic

Modified Classification: None

Reason for modified classification: None

Interpretation Status: PROVISIONAL

Date interpretation saved: 2024 Oct 07, 2:06 pm

Disease: X-linked retinoschisis

Mode of Inheritance: X-linked inheritance (recessive)

Specification Document: None

Evidence Summary

The NM_000330.4(RS1):c.326+1G>A variant is a canonical splice site variant in intron 4, located 1 nucleotide after exon 4. This variant occurs at a canonical splice site in intron 4 and is predicted to disrupt splicing and induce skipping of exon 5, which is expected to disrupt a critical functional domain in RS1 (PVS1). This variant is absent from hemizygous individuals in gnomAD v4.1.0 (PM2_Supporting). The variant has been reported to segregate with retinal dystrophy through 1 meiosis in a family of two affected brothers. (PP1; PMID: 15937075). This variant has been reported in at least 2 apparently unrelated probands diagnosed with XLRS (PMID: 9618178, 15937075, 29099798, 30025115, 34822951, PS4_supporting). In summary, this variant is classified as pathogenic for X-linked retinoschisis based on the ACMG/AMP criteria applied, as specified by the ClinGen X-linked IRD VCEP: PVS1, PM2_supporting, PS4_supporting, and PP1. (VCEP specifications version PILOT; date of approval 07/11/2024).

Criteria meeting an evaluation strength

B/P	Criteria	Criteria Descriptions	Modified	Status	Explanation
✓	PVS1	Predicted null variant in a gene where LOF is a known mechanism of disease	No	met	This variant occurs at a canonical splice site in intron 4 and is predicted to disrupt splicing and induce skipping of exon 5, which is expected to disrupt a critical functional domain in RS1 (PVS1).
✓	PP1	Cosegregation with disease in multiple affected family members	No	met	The variant has been reported to segregate with retinal dystrophy through 1 meiosis in a family two affected brothers. (PP1; PMID: 15937075).
✓	PM2	Absent in population databases	Yes	PM2_supporting	This variant is absent from hemizygous individuals in gnomAD v4.1.0 (PM2_Supporting).
✓	PS4	Prevalence in affecteds statistically increased over controls	Yes	PS4_supporting	This variant has been reported in at least 2 apparently unrelated probands diagnosed with XLRS (PMID: 9618178, 15937075, 29099798, 30025115, 34822951).

Criteria evaluated as 'Not met'

B/P	Criteria	Criteria Descriptions	Modified	Status	Explanation

Criteria 'Not yet evaluated'

B/P	Criteria	Criteria Descriptions	Modified	Status	Explanation
○	BA1	Allele frequency greater than 5% in a population database	N/A	Not Evaluated	
○	BS1	MAF is too high for disorder	N/A	Not Evaluated	
○	BS2	Observation in controls inconsistent with disease penetrance	N/A	Not Evaluated	
○	BS3	Well-established functional studies show no deleterious effect	N/A	Not Evaluated	
○	BS4	Non-segregation with disease	N/A	Not Evaluated	
○	PS1	Same amino acid change as an established pathogenic variant	N/A	Not Evaluated	
○	PS2	De novo (paternity and maternity confirmed)	N/A	Not Evaluated	
○	PS3	Well-established functional studies show a deleterious effect	N/A	Not Evaluated	

B/P	Criteria	Criteria Descriptions	Modified	Status	Explanation
	**PM1**	Mutational hot spot or well-studied functional domain without benign variation	N/A	Not Evaluated	
	**PM3**	For recessive disorders, detected in trans with a pathogenic variant	N/A	Not Evaluated	
	**PM4**	Protein length changing variant	N/A	Not Evaluated	
	**PM5**	Novel missense change at an amino acid residue where a different pathogenic missense change has been seen before	N/A	Not Evaluated	
	**PM6**	De novo (without paternity & maternity confirmed)	N/A	Not Evaluated	
	**BP1**	Missense in gene where primarily truncating cause disease	N/A	Not Evaluated	
	**BP2**	Observed in trans with path. variant for dominant disorder or cis any inheritance	N/A	Not Evaluated	
	**BP3**	In-frame indels in repeat w/out known function	N/A	Not Evaluated	
	**BP4**	Multiple lines of computational evidence suggest no impact on gene /gene product	N/A	Not Evaluated	
	**BP5**	Found in case with an alternate cause	N/A	Not Evaluated	

B/P	Criteria	Criteria Descriptions	Modified	Status	Explanation
○	BP6	Reputable source w/out shared data = benign	N/A	Not Evaluated	
○	BP7	Silent variant predicted with no splice impact	N/A	Not Evaluated	
○	PP2	Missense in gene with low rate of benign missense variants and path. missenses common	N/A	Not Evaluated	
○	PP3	Multiple lines of computational evidence support a deleterious effect on the gene /gene product	N/A	Not Evaluated	
○	PP4	Patient's phenotype or FH highly specific for gene	N/A	Not Evaluated	
○	PP5	Reputable source = pathogenic	N/A	Not Evaluated	

Print PDF

Evaluation Summary

NM_000330.4(RS1):c.472G>A (p.Asp158Asn)

ClinVar VariationID: 98969

ClinGen Allele Registry ID: CA226749

Interpretation owner: X-linked Inherited Retinal Disease

Auto-calculated Classification: Uncertain significance - insufficient evidence

Modified Classification: Likely benign

Reason for modified classification: The ClinGen XLIRD VCEP recommends calculation of the final variant classification using a points system rather than the standard auto-calculation (VCEP specifications version PILOT; date of approval 09/05/2023).

Interpretation Status: PROVISIONAL

Date interpretation saved: 2024 Oct 07, 3:42 pm

Disease: X-linked retinoschisis

Mode of Inheritance: X-linked inheritance

Specification Document: None

Evidence Summary

The NM_000330.4(RS1):c.472G>A variant is a missense variant encoding the substitution of Aspartic acid with Asparagine at position 158. This variant is present in gnomAD v.4.1.0 at a frequency of 0.000005012 among hemizygous individuals, with 2 variant alleles / 399058 total hemizygous alleles, which is between the ClinGen X-linked IRD VCEP PM2_Supporting and BS1 threshold of 0.000002 to 0.00002 and fails to meet these criteria. The computational predictor REVEL gives a score of 0.395, which is between the ClinGen X-linked IRD VCEP threshold of 0.664 to 0.290 and does not predict a damaging effect on RS1 function. Additionally, the splicing impact predictor SpliceAI gives a score of 0.00, which is below the ClinGen X-linked IRD VCEP recommended threshold of ≥0.2 and does not strongly predict an impact on splicing. Collectively, both BP4 and PP3 codes do not apply. EBNA293 cells exogenously expressing the variant exhibit no loss of RS1 secretion into the medium relative to the wild-type control (PMID: 17525175). DOI: 10.5772/24453 predicted this change to have a mild effect. The Cspec does not allow the use of this code, the BS3 code is unmet. DOI: 10.5772/24453 predicted this change to have a mild effect. Sieving et al. report on this variant to be polymorphism (PMID: 10703138, BS2). In summary, this variant is classified as likely benign for X-linked retinoschisis based on the ACMG/AMP criteria applied, as specified by the ClinGen X-linked IRD VCEP: BS2 (VCEP specifications version PILOT; date of approval 11/02/2024).

Criteria meeting an evaluation strength

B/P	Criteria	Criteria Descriptions	Modified	Status	Explanation
✔	BS2	Observation in controls inconsistent with disease penetrance	No	met	Sieving et al. report on this variant to be polymorphism (PMID: 10703138, BS2).

Criteria evaluated as 'Not met'

B/P	Criteria	Criteria Descriptions	Modified	Status	Explanation
✖	BS1	MAF is too high for disorder	N/A	Not Met	This variant is present in gnomAD v.4.1.0 at a frequency of 0.000005012 among hemizygous individuals,<br>with 2 variant alleles / 399058 total hemizygous alleles, which is between the ClinGen X-linked IRD<br>VCEP PM2_Supporting and BS1 threshold of 0.000002 to 0.00002 and fails to meet these criteria.

B/P	Criteria	Criteria Descriptions	Modified	Status	Explanation
✖	**BS3**	Well-established functional studies show no deleterious effect	N/A	Not Met	EBNA293 cells exogenously expressing the variant exhibit no loss of RS1 secretion into the medium relative to the wild-type control (PMID: 17525175). DOI: 10.5772/24453 predicted this change to have a mild effect. The Cspec CSpec does not allow the use of this code, the BS3 code is unmet.
✖	**PM2**	Absent in population databases	N/A	Not Met	This variant is present in gnomAD v.4.1.0 at a frequency of 0.000005012 among hemizygous individuals,<br>with 2 variant alleles / 399058 total hemizygous alleles, which is between the ClinGen X-linked IRD<br>VCEP PM2_Supporting and BS1 threshold of 0.000002 to 0.00002 and fails to meet these criteria.

B/P	Criteria	Criteria Descriptions	Modified	Status	Explanation
✖	BP4	Multiple lines of computational evidence suggest no impact on gene /gene product	N/A	Not Met	The computational predictor REVEL gives a score of 0.395, which is between the ClinGen X-linked IRD VCEP threshold of 0.664 to 0.290 and does not predict a damaging effect on RS1 function. Additionally, the splicing impact predictor SpliceAI gives a score of 0.00, which is below the ClinGen X-linked IRD VCEP recommended threshold of ≥0.2 and does not strongly predict an impact on splicing. Collectively, both BP4 and PP3 codes do not apply.
✖	PP3	Multiple lines of computational evidence support a deleterious effect on the gene /gene product	N/A	Not Met	The computational predictor REVEL gives a score of 0.395, which is between the ClinGen X-linked IRD VCEP threshold of 0.664 to 0.290 and does not predict a damaging effect on RS1 function. Additionally, the splicing impact predictor SpliceAI gives a score of 0.00, which is below the ClinGen X-linked IRD VCEP recommended threshold of ≥0.2 and does not strongly predict an impact on splicing. Collectively, both BP4 and PP3 codes do not apply.

Criteria 'Not yet evaluated'

B/P	Criteria	Criteria Descriptions	Modified	Status	Explanation
○	BA1	Allele frequency greater than 5% in a population database	N/A	Not Evaluated	
○	PVS1	Predicted null variant in a gene where LOF is a known mechanism of disease	N/A	Not Evaluated	
○	BS4	Non-segregation with disease	N/A	Not Evaluated	
○	PS1	Same amino acid change as an established pathogenic variant	N/A	Not Evaluated	
○	PS2	De novo (paternity and maternity confirmed)	N/A	Not Evaluated	
○	PS3	Well-established functional studies show a deleterious effect	N/A	Not Evaluated	
○	PS4	Prevalence in affecteds statistically increased over controls	N/A	Not Evaluated	
○	PM1	Mutational hot spot or well-studied functional domain without benign variation	N/A	Not Evaluated	
○	PM3	For recessive disorders, detected in trans with a pathogenic variant	N/A	Not Evaluated	
○	PM4	Protein length changing variant	N/A	Not Evaluated	

B/P	Criteria	Criteria Descriptions	Modified	Status	Explanation
○	PM5	Novel missense change at an amino acid residue where a different pathogenic missense change has been seen before	N/A	Not Evaluated	
○	PM6	De novo (without paternity & maternity confirmed)	N/A	Not Evaluated	
○	BP1	Missense in gene where primarily truncating cause disease	N/A	Not Evaluated	
○	BP2	Observed in trans with path. variant for dominant disorder or cis any inheritance	N/A	Not Evaluated	
○	BP3	In-frame indels in repeat w/out known function	N/A	Not Evaluated	
○	BP5	Found in case with an alternate cause	N/A	Not Evaluated	
○	BP6	Reputable source w/out shared data = benign	N/A	Not Evaluated	
○	BP7	Silent variant predicted with no splice impact	N/A	Not Evaluated	
○	PP1	Cosegregation with disease in multiple affected family members	N/A	Not Evaluated	
○	PP2	Missense in gene with low rate of benign missense variants and path. missenses common	N/A	Not Evaluated	

B/P	Criteria	Criteria Descriptions	Modified	Status	Explanation
○	PP4	Patient's phenotype or FH highly specific for gene	N/A	Not Evaluated	
○	PP5	Reputable source = pathogenic	N/A	Not Evaluated	

Print PDF

Evaluation Summary

NM_000330.4(RS1):c.478C>T (p.Arg160Cys)

ClinVar VariationID: 1103437

ClinGen Allele Registry ID: CA10360634

Interpretation owner: X-linked Inherited Retinal Disease

Auto-calculated Classification: Uncertain significance - insufficient evidence

Modified Classification: Likely benign

Reason for modified classification: The ClinGen XLIRD VCEP recommends calculation of the final variant classification using a points system rather than the standard auto-calculation (VCEP specifications version PILOT; date of approval 09/05/2023).

Interpretation Status: PROVISIONAL

Date interpretation saved: 2024 Oct 07, 3:22 pm

Disease: X-linked retinoschisis

Mode of Inheritance: X-linked inheritance

Specification Document: None

Evidence Summary

The NM_000330.4(RS1):c.478C>T variant is a missense variant encoding the substitution of Arginine with Cystine at position 160. This variant is present in gnomAD v.4.1.0 at a frequency of 0.00002772 among hemizygous individuals, with 11 variant alleles / 396847 total alleles, which is higher than the ClinGen X-linked IRD VCEP BS1 threshold of >>0.00002 (BS1). The computational predictor REVEL gives a score of 0.391, which is between the ClinGen X-linked IRD VCEP threshold of 0.664 to 0.290 and does not predict a damaging effect on RS1 function. Additionally, the splicing impact predictor SpliceAI gives a score of 0.01, which is below the ClinGen X-linked IRD VCEP recommended threshold of ≥0.2 and does not strongly predict an impact on splicing. Collectively, both BP4 and PP3 codes do not apply. In summary, this variant is classified as likely benign for X-linked retinoschisis based on the ACMG/AMP criteria applied, as specified by the ClinGen X-linked IRD VCEP: BS1. VCEP specifications version PILOT; date of approval 07/11/2024.

Criteria meeting an evaluation strength

B/P	Criteria	Criteria Descriptions	Modified	Status	Explanation
✔	BS1	MAF is too high for disorder	No	met	This variant is present in gnomAD v.4.1.0 at a frequency of 0.00002772 among hemizygous individuals, with 11 variant alleles / 396847 total alleles, which is higher than the ClinGen X-linked IRD VCEP BS1 threshold of >>0.00002 (BS1).

Criteria evaluated as 'Not met'

B/P	Criteria	Criteria Descriptions	Modified	Status	Explanation
✖	BP4	Multiple lines of computational evidence suggest no impact on gene /gene product	N/A	Not Met	The computational predictor REVEL gives a score of 0.391, which is between the ClinGen X-linked IRD VCEP threshold of 0.664 to 0.290 and does not predict a damaging effect on RS1 function. Additionally, the splicing impact predictor SpliceAI gives a score of 0.01, which is below the ClinGen X-linked IRD VCEP recommended threshold of ≥0.2 and does not strongly predict an impact on splicing. Collectively, both BP4 and PP3 codes do not apply.

B/P	Criteria	Criteria Descriptions	Modified	Status	Explanation
✖	PP3	Multiple lines of computational evidence support a deleterious effect on the gene /gene product	N/A	Not Met	The computational predictor REVEL gives a score of 0.391, which is between the ClinGen X-linked IRD VCEP threshold of 0.664 to 0.290 and does not predict a damaging effect on RS1 function. Additionally, the splicing impact predictor SpliceAI gives a score of 0.01, which is below the ClinGen X-linked IRD VCEP recommended threshold of ≥0.2 and does not strongly predict an impact on splicing. Collectively, both BP4 and PP3 codes do not apply.

Criteria 'Not yet evaluated'

B/P	Criteria	Criteria Descriptions	Modified	Status	Explanation
○	BA1	Allele frequency greater than 5% in a population database	N/A	Not Evaluated	
○	PVS1	Predicted null variant in a gene where LOF is a known mechanism of disease	N/A	Not Evaluated	
○	BS2	Observation in controls inconsistent with disease penetrance	N/A	Not Evaluated	

B/P	Criteria	Criteria Descriptions	Modified	Status	Explanation
	BS3	Well-established functional studies show no deleterious effect	N/A	Not Evaluated	
	BS4	Non-segregation with disease	N/A	Not Evaluated	
	PS1	Same amino acid change as an established pathogenic variant	N/A	Not Evaluated	
	PS2	De novo (paternity and maternity confirmed)	N/A	Not Evaluated	
	PS3	Well-established functional studies show a deleterious effect	N/A	Not Evaluated	
	PS4	Prevalence in affecteds statistically increased over controls	N/A	Not Evaluated	
	PM1	Mutational hot spot or well-studied functional domain without benign variation	N/A	Not Evaluated	
	PM2	Absent in population databases	N/A	Not Evaluated	
	PM3	For recessive disorders, detected in trans with a pathogenic variant	N/A	Not Evaluated	
	PM4	Protein length changing variant	N/A	Not Evaluated	

B/P	Criteria	Criteria Descriptions	Modified	Status	Explanation
○	PM5	Novel missense change at an amino acid residue where a different pathogenic missense change has been seen before	N/A	Not Evaluated	
○	PM6	De novo (without paternity & maternity confirmed)	N/A	Not Evaluated	
○	BP1	Missense in gene where primarily truncating cause disease	N/A	Not Evaluated	
○	BP2	Observed in trans with path. variant for dominant disorder or cis any inheritance	N/A	Not Evaluated	
○	BP3	In-frame indels in repeat w/out known function	N/A	Not Evaluated	
○	BP5	Found in case with an alternate cause	N/A	Not Evaluated	
○	BP6	Reputable source w/out shared data = benign	N/A	Not Evaluated	
○	BP7	Silent variant predicted with no splice impact	N/A	Not Evaluated	
○	PP1	Cosegregation with disease in multiple affected family members	N/A	Not Evaluated	
○	PP2	Missense in gene with low rate of benign missense variants and path. missenses common	N/A	Not Evaluated	

B/P	Criteria	Criteria Descriptions	Modified	Status	Explanation
○	PP4	Patient's phenotype or FH highly specific for gene	N/A	Not Evaluated	
○	PP5	Reputable source = pathogenic	N/A	Not Evaluated	

Print PDF
