# Supplementary material for: Development of RS1-specific ACMG/AMP variant classification criteria with pilot variant curation
Source: Hum Genet. 2026 Sep 4;145(1):75. doi: 10.1007/s00439-026-02871-0 (PMC13545035; doi:10.1007/s00439-026-02871-0)
Supplement: Supplementary file 3 [file 439_2026_2871_MOESM1_ESM.docx]

**Supp Figure 1: Area under the curve (AUC) scores for 11 classifiers.** Performance of 11 classifiers for 42 pathogenic and 24 benign annotated ClinVar variants using receiver operating characteristic (ROC) curve analysis and area under the curve (AUC) scores as previously described (Brock et al 2024). The best performing classifier was REVEL.

Brock DC, Wang M, Hussain HMJ, et al. Comparative analysis of in-silico tools in identifying pathogenic variants in dominant inherited retinal diseases. Hum Mol Genet. 2024 May 18;33(11):945-957. PMID: 38453143.
