# Supplementary material for: Development of RS1-specific ACMG/AMP variant classification criteria with pilot variant curation
Source: Hum Genet. 2026 Sep 4;145(1):75. doi: 10.1007/s00439-026-02871-0 (PMC13545035; doi:10.1007/s00439-026-02871-0)
Supplement: Supplementary file 4 [file 439_2026_2871_MOESM5_ESM.docx]

**Supplementary table 1**: *RS1* PS2 points awarded per de novo occurrence

| Points per de novo occurrence for X-linked IRD genes | *RS1* points per Proband | |
| --- | --- | --- |
|  | Confirmed de novo with confirmed maternity | Assumed de novo with assumed maternity |
| Phenotype highly specific for gene as per PP4 criteria | 2 | 1 |
| Phenotype consistent with gene but not highly specific | 1 | 0.5 |

**Supplementary Table 2**: All *RS1* pilot variants

Locations are noted in transcript GenBank: NM_000330.4. Abbreviations: N/A is not available; VUS is variant of uncertain significance

| **Coding DNA** | **Protein** | **Exon** | **ClinVar ID or Allele Registry ID** | **Allele frequencies** | **ClinVar Classification** | **Type** | **Exon/ Intron** | **Pilot Classification** | **Scoring system** | **Codes used** |
| --- | --- | --- | --- | --- | --- | --- | --- | --- | --- | --- |
| c.1A>T | p.Met1Leu | 1 | 488837 | -- | Pathogenic | Start loss | - | Pathogenic | 14 | PM2_supp, PVS1, PP4, PP1_strong |
| c.7C>G | p.Arg3Gly | 1 | 757382 | 0.0013 | Benign | Missense | 1 | Benign | -9 | BA1, BP4 |
| c.26del | p.Leu9Cysfs*117 | 1 | CA2695231613 | -- | Absent | Deletion | 1 | Pathogenic | 14 | PVS1, PM2_supp, PP4, PP1_str |
| c.33_36del | p.(Leu11Phefs*114) | 1 | 98944 | -- | Pathogenic | Deletion | 1 | Pathogenic | 12 | PVS1, PM2_supp, PP1_mod, PS4_supp |
| c.35T>A | p.Leu12His | 1 | 98946 | -- | Likely Pathogenic | Missense | 1 | VUS | 3 | PS3_supp, PS4_supp, PM2_supp |
| c.37C>T | p.Leu13Phe | 1 | 1421996 | 1E-05 | VUS | Missense | 1 | VUS | 1 | PS3_supp |
| c.53-713_78+266del | --- | exon 2 del | 1048161 | -- | Likely pathogenic | Exon deletion | 2 | Pathogenic | 13 | PVS1, PM2_supp, PP1_Strong |
| c.61G>C | p.Gly21Arg | 2 | 1063524 | -- | VUS | Missense | 2 | VUS | 1 | PM2_supp |
| c.68C>A | p.Ser23Ter | 2 | 99020 | -- | Not provided | Nonsense | 2 | Pathogenic | 10 | PVS1, PM2_supp, PS4_supp |
| c.69G>A | p.Ser23= | 2 | 1671968 | 8E-06 | Likely benign | Synonymous | 2 | Likely benign | -2 | BP7, BP4 |
| c.76G>A | p.Glu26Lys | 2 | 741219 | 0.0004 | Benign | Missense | 2 | Benign | -8 | BA1 |
| c.76G>T | p.Glu26Ter | 2 | 99021 | -- | Not provided | Nonsense | 2 | Likely pathogenic | 9 | PVS1, PM2_supp |
| c.78+1G>A | --- | i2 | 99022 | -- | Pathogenic | Splice site | i2 | Pathogenic | 11 | PVS1, PM2_supp, PP4, PS4_supp (1) |
| c.78+5G>A | --- | i2 | CA2695231369 | -- | Absent | intron +5 | i2 | Likely pathogenic | 6 | PM2_supp, PP3, PS4_mod, PS1_mod |
| c.88G>A | p.Glu30Lys | 3 | 722780 | 0.0002 | Benign | Missense | 3 | Likely benign | -4 | BS1 |
| c.96dup | p.Trp33LeufsTer53 | 3 | CA2580650463 | -- | Absent | Duplication | 3 | Pathogenic | 11 | PVS1, PM2_supp, PP4, PS4_supp |
| c.131dup | p.Gly45fs | 3 | 1687568 | -- | Likely Pathogenic | Duplication | 3 | Likely pathogenic | 9 | PVS1, PM2_supp |
| c.145C>T | p.Leu49= | 3 | 1570461 | -- | Likely benign | Synonymous | 3 | Likely benign | -1 | PM2_supp, BP7, BP4 |
| c.149G>A | p.Trp50Ter | 3 | 1419115 | -- | Pathogenic | Nonsense | 3 | Pathogenic | 10 | PVS1, PM2_supp, PP4 |
| c.150G>T | p.Trp50Cys | 3 | 1164535 | 0.0002 | Benign | Missense | 3 | Likely benign | -4 | BS1 |
| c.170T>C | p.Leu57Ser | 3 | 423184 | -- | VUS | Missense | 3 | VUS | 2 | PM2_supp, PP3 |
| c.176G>A | p.Cys59Tyr | 3 | 866404 | -- | Likely pathogenic | Missense | 3 | Likely pathogenic | 8 | PM2_supp, PM1_strong, PM5, PS4_supp, PP4 |
| c.176G>C | p.Cys59Ser | 3 | CA412376053 | -- | Absent | Missense | 3 | Pathogenic | 10 | PM2_supp, PP3_mod, PS1, PS3_supp, PS4_supp, PP4 |
| c.184+9C>T | --- | i3 | 755144 | 1E-05 | Benign | Missense | 3 | Likely benign | -2 | BP4, BP7 |
| c.203C>G | p.Pro68Arg | 4 | CA412372976 | -- | Absent | Missense | 4 | VUS | 5 | PM2_supp, PP3_strong |
| c.214G>A | p.Glu72Lys | 4 | 9888 | 3E-06 | Pathogenic/Likely pathogenic | Missense | 4 | Pathogenic | 14 | PP3_mod, PP1_strong, PP4, PS4, PS3_supporting, PM1_moderate |
| c.221_237delinsTCCCCTG  ACCGGGTTAGAGT | p.Gly74_Asp79delinsValProTer | 4 | 449509 | -- | Pathogenic | indel | 4 | Pathogenic | 12 | PVS1, PM2_supp, PP1_mod, PP4. |
| c.227T>A | p.Val76Asp | 4 | 1066419 | -- | Pathogenic | Missense | 4 | Pathogenic | 11 | PM2_supp, PP3_strong, PP1_strong, PP4_mod |
| c.253_255del | p.Asn85del | 4 | 98921 | -- | Conflicting | In-frame deletion | 4 | VUS | 4 | PM2_supp, PM4_mod, PS4_supp |
| c.258G>A | p.Pro86Pro | 4 | 791378 | 0.0007 | Benign | Missense | 4 | Benign | -10 | BA1, BP4_supp, BP7. |
| c.264G>C | p.Gln88His | 4 | 730036 | 0.0003 | Benign | Missense | 4 | Likely benign | -6 | BA1, PP3_mod |
| c.276G>C | p.Trp92Cys | 4 | 98925 | -- | Pathogenic | Missense | 4 | Pathogenic | 10 | PM2_supp, PP3_strong, PP1_mod, PP4, PS4_mod |
| c.295A>G | p.Asn99Asp | 4 | 710170 | 0.0027 | Benign | Missense | 4 | Benign | -8 | BA1 |
| c.304C>T | p.Arg102Trp | 4 | 9887 | 8e-6 | Pathogenic/Likely pathogenic | Missense | 4 | Pathogenic | 13 | PP3_strong, PP1_strong, PP4, PS4 |
| c.326+1G>A | --- | i4 | 98936 | -- | Not provided | Splice site | i4 | Pathogenic | 11 | PM2_supporting, PVS1, PP1, PS4_supp |
| c.326+14G>A | --- | i4 | 1600897 | 0.0003 | Benign | intron | i4 | Benign | -10 | BA1, BP4_supp, BP7 |
| c.330T>C | p.Cys110= | 5 | 98940 | 0.0143 | Benign | Synonymous | 5 | Benign | -10 | BA1, BP4, BP7 |
| c.337C>T | p.Leu113Phe | 5 | 98943 | -- | Pathogenic/Likely pathogenic | Missense | 5 | Likely pathogenic | 8 | PM2_supp, PP3_mod, PS3_supp, PS4 |
| c.421C>G | p.Arg141Gly | 5 | 98958 | -- | Pathogenic | Missense | 5 | Likely pathogenic | 8 | PM2_supp, PP3_mod, PS3_supp, PP1, PM5_mod, PS4_supp |
| c.426T>C | p.Cys142Cys | 5 | 98961 | -- | Not provided | Synonymous | 5 | Likely benign | -1 | PM2_supporting, BP4, and BP7 |
| c.433G>A | p.Asp145Asn | 5 | 798785 | -- | Likely benign | Missense | 5 | VUS | 0 | --- |
| c.441G>A | p.Trp147Ter | 5 | CA412371717 | -- | Absent | Nonsense | 5 | Pathogenic | 11 | PM2_supp, PVS1, PP1, and PS4_supp |
| c.472G>A | p.Asp158Asn | 5 | 98969 | 5e-06 | Conflicting | Missense | 5 | Likely benign | -4 | BS2 |
| c.478C>T | p.Arg160Cys | 5 | 1103437 | 3E-05 | Likely benign | Missense | 5 | Likely benign | -4 | BS1 |
| c.498C>A | p.Tyr166Ter | 5 | 370754 | -- | Pathogenic/Likely pathogenic | Nonsense | 5 | Pathogenic | 10 | PM2_supp, PVS1, PS4_supp |
| c.520del | p.Arg174fs | 5 | 279886 | -- | Pathogenic | Deletion | 5 | Pathogenic | 10 | PM2_supp, PVS1, PS4_supp |
| c.533G>C | p.Gly178Ala | 6 | CA412371717 | -- | Absent | Missense | 6 | Likely pathogenic | 6 | PM2_supp, PP3_strong, PP1 |
| c.548C>T | p.Thr183Ile | 6 | 736967 | 0.0013 | Benign | Missense | 6 | Benign | -8 | BA1 |
| c.576C>T | p.Pro192= | 6 | 255840 | 0.0004 | Benign/Likely benign | Synonymous | 6 | Benign | -10 | BA1, BP4, and BP7. |
| c.583_585dupATC | p.Ile195dup | 6 | 68077 | -- | VUS | In-frame duplication | 6 | VUS | 5 | PM2_supp, PM4_mod, PS3_supp, PP4. |
| c.589C>T | p.Arg197Cys | 6 | 98996 | -- | Pathogenic | Missense | 6 | Pathogenic | 11 | PM2_supp, PP3_strong, PP1_mod, PP4_mod, PS4_mod |
| c.647T>c | p.Leu216Pro | 6 | 99014 | -- | Pathogenic | Missense | 6 | Likely pathogenic | 8 | PM2_supp, PP3_strong, PP4, PS4_mod |
| c.668G>A | p.Cys223Tyr | 6 | 372497 | -- | Pathogenic | Missense | 6 | Pathogenic | 10 | PM2_supp, PP3_strong, PP1_supp, PP4, PM5, PS4_mod |
| c.673T>C | p.Ter225Arg | 6 | 371642 | -- | Likely Pathogenic | Stop-loss | 6 | VUS | 5 | PM2_supporting, PM4_strong |

**Supp Table 3: Summary of pilot variant classification**

| **Classification** | **ClinVar number** | **VCEP Number** | **Detail** |
| --- | --- | --- | --- |
| Pathogenic (P) | 12 | 20 | 10 remained P  2 P became LP  1 LP became P  3 P/LP reclassified as P  **2 not provided reclassified as P**  **4 absent from ClinVar classified as P** |
| Pathogenic/Likely Pathogenic (P/LP) | 4 | - | 3 became P  1 became LP |
| Likely Pathogenic (LP) | 5 | 8 | 2 remained LP  **2 LP became VUS**  **1 LP became P**  2 P became LP  1 P/LP became LP  **1 not provided became LP**  **2 absent from ClinVar classified as LP** |
| Variant of uncertain significance (VUS) | 4 | 9 | 4 VUS remained VUS  2 LP became VUS  1 LB became VUS  1 conflicting became VUS  1 absent from ClinVar classified as VUS |
| Likely Benign (LB) | 4 | 9 | 3 LB remained LB  1 LB became a VUS  4 B became LB  1 conflicting became LB  1 not provided became LB |
| Benign/Likely Benign (B/LB) | 1 | - | 1 B/LB became B |
| Benign (B) | 11 | 8 | 7 remained B  4 became LB  1 B/LB became B |
| Conflicting (1 LP/VUS, 1 VUS/LB) | 2 | - | 1 became VUS  1 became LB |
| Not provided | 4 | - | 2 became P  1 became LP  1 became LB |
| Absent from ClinVar | - | 7 | 4 became P  2 became LP  1 became VUS |
